# Preparation and Uses of Chlorinated Glycerol Derivatives

**DOI:** 10.3390/molecules25112511

**Published:** 2020-05-28

**Authors:** Anna Canela-Xandri, Mercè Balcells, Gemma Villorbina, Paul Christou, Ramon Canela-Garayoa

**Affiliations:** 1 Department of Chemistry, University of Lleida-Agrotecnio Centre and DBA center, Av. Alcalde Rovira Roure, 191, 25198 Lleida, Spain; acanelxa@quimica.udl.cat (A.C.-X.); merce.balcells@udl.cat (M.B.); gemma.villorbina@udl.cat (G.V.); 2 Department of Crop and Forest Sciences, University of Lleida-Agrotecnio Center, Av. Rovira Roure 191, 25198 Lleida, Spain; christou@pvcf.udl.cat; 3 ICREA, Catalan Institute for Research and Advanced Studies, Passeig Lluıís Companys 23, 08010 Barcelona, Spain

**Keywords:** bioeconomy, chlorohydrins, epichlorohydrin, hydrocloride derivatives, glycerol

## Abstract

Crude glycerol (C_3_H_8_O_3_) is a major by-product of biodiesel production from vegetable oils and animal fats. The increased biodiesel production in the last two decades has forced glycerol production up and prices down. However, crude glycerol from biodiesel production is not of adequate purity for industrial uses, including food, cosmetics and pharmaceuticals. The purification process of crude glycerol to reach the quality standards required by industry is expensive and dificult. Novel uses for crude glycerol can reduce the price of biodiesel and make it an economical alternative to diesel. Moreover, novel uses may improve environmental impact, since crude glycerol disposal is expensive and dificult. Glycerol is a versatile molecule with many potential applications in fermentation processes and synthetic chemistry. It serves as a glucose substitute in microbial growth media and as a precursor in the synthesis of a number of commercial intermediates or fine chemicals. Chlorinated derivatives of glycerol are an important class of such chemicals. The main focus of this review is the conversion of glycerol to chlorinated derivatives, such as epichlorohydrin and chlorohydrins, and their further use in the synthesis of additional downstream products. Downstream products include non-cyclic compounds with allyl, nitrile, azide and other functional groups, as well as oxazolidinones and triazoles, which are cyclic compounds derived from ephichlorohydrin and chlorohydrins. The polymers and ionic liquids, which use glycerol as an initial building block, are highlighted, as well.

## 1. Introduction

Society currently faces the twin challenge of resource depletion and waste accumulation. This challenge leads to a rapid increase in the costs of raw materials and waste disposal, which is subject to restrictive and burdensome legislation. Thus, environmental pollution and waste accumulation are key factors in valorising biomass in the transition to a low-carbon economy society and the decarbonization of carbon-intensive sectors. An essential component of this valorization is the “zero-waste” concept [1,2,3].

Efficient use of biomass as a source of fine chemicals will play an important role in sustainable development and mitigating global warming [4,5]. 

Biomass can also be used to obtain biofuels such as bioethanol, biomethane and biodiesel [6,7,8,9]. Replacing fossil fuels with renewable resources will lead to the reduction of waste accumulation by revaluating industrial by-products and reducing resource depletion [10,11]. Moreover, rising crude oil prices have stimulated interest in developing alternative renewable biofuels in the recent past. More recently, however, oil prices have collapsed; it is unclear what the impact of this might be on the continuing use of biofuels, particularly if oil prices remain low indefinitely.

Biodiesel can be produced from many renewable sources. These include vegetable oils and animal fats. The process usually involves transesterification of acylglycerides into fatty acid methyl esters (FAME), with glycerol (C_3_H_8_O_3_) as the major by-product. On a molar basis, one mole of glycerol is produced for every three moles of FAME. Hence, 10% of the initial acylglycerides are roughly converted to glycerol. Crude glycerol resulting from the biodiesel industry becomes, itself, a source of biomass. Glycerol is a versatile molecule with many potential applications [12,13,14,15,16,17,18]. In fact, novel uses of glycerol may be instrumental in making biodiesel a competitive alternative fuel to petroleum-based fuels.

Pure glycerol is physiologically innocuous, and it is currently used in a large variety of applications, primarily in the cosmetic, food and pharmaceutical industries. However, the use of glycerol in these industries is limited by strict physical, chemical and biological requirements. Requirements that crude glycerol resulting from biodiesel production does not meet [19]. In 2011, it was estimated that two million tons (or just 40%) of a total of 5.1 million tons of glycerol were used [19]. However, the volume of glycerol has been steadily increasing because global biodiesel production has been growing in recent years. It is estimated that the biodiesel production could reach 41 Mm^3^ in 2022 [19], considering the 9.3% year increase in world glycerol production between 2008 and 2012. Thus, the glycerol market is becoming a bottleneck on biodiesel production [19,20]. 

**Figure 1:**
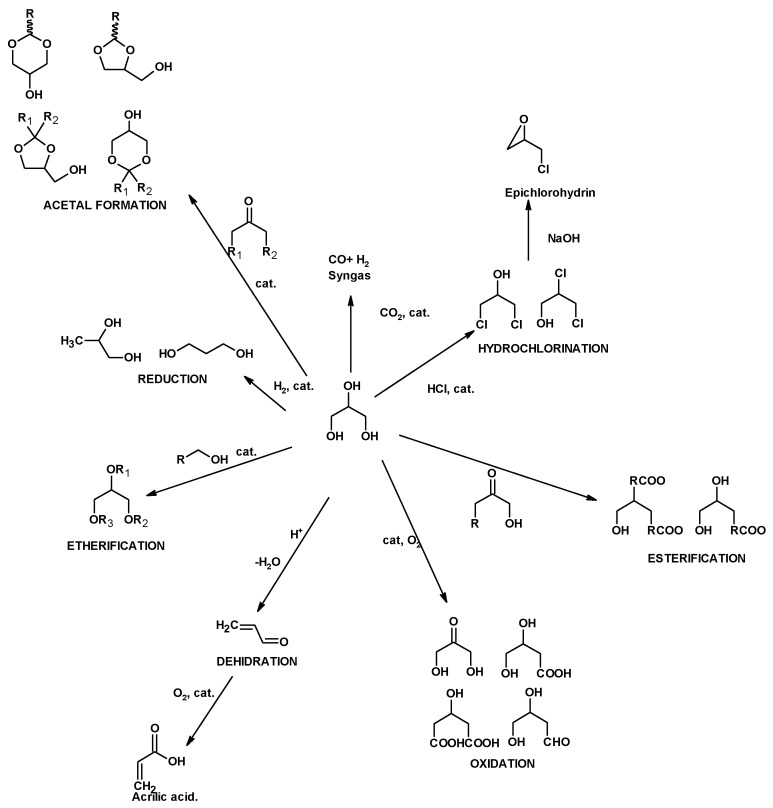
Reactions in which glycerol is used as a building block to make more-complex molecules [51].

Glycerol can be used as both an energy source and a platform chemical. Direct pyrolysis [21], direct combustion and hydrogen production are usual processes where crude glycerol can be used as energy source [22]. However, glycerol high viscosity hinders flow spraying, pumping and flame stability. Uncontrolled burning produces acrolein (2-propenal), an unsaturated aldehyde with severe detrimental effects on the human health [19]. Moreover, hydrogen preparation from crude glycerol involves high production costs [23,24,25,26]. The transformation of glycerol into fine chemicals can be performed by chemical and biological processes. However, most of these glycerol derivatives are currently produced by expensive processes, and therefore their utilization on an industrial scale is still limited [27]. Glycerol can be converted into more complex intermediates and products through a number of different chemical reactions. Figure 1 shows the most reactions in which glycerol can be involved as a building block [28]. Despite the large number of theoretical possibilities, in practice, there are two possible areas to use up the large amount of glycerol produced by the biodiesel industry: as feedstock for commodity chemicals [29,30,31,32,33,34], or for producing oxygenated additives for fuels [27,35,36,37,38]. As an example, glycerol can be thermochemically converted to propylene glycol [29,30,31] and acrolein; the latter can be oxidized to acrylic acid [39,40,41]. Glycerol can be esterified to acylglycerides and glycerol carbonates [42]. It can also be used to prepare chlorinated derivatives. Synthetic pathways to chlorhydrines have been described, many of them leading to a mixture of isomers [32,34,43,44,45]. These products exhibit some degree of toxicity [46,47,48,49]. As an alternative, the authors’ research group has described the synthesis of chlorohydrin esters by using crude glycerol and different fatty acids. These esters are less volatile than the corresponding chlorohydrins, which, in principle, reduce their toxicity as chemical reagents [50]. 

Glycerol is also involved in biological transformations. Crude glycerol is a suitable feedstock in microbial fermentation. It has been used for the production of succinic acid, using the bacterium *Anaerobiospirillum succiniciproducens* [52] and citric acid, using the yeast *Yarrowia lipolytica.* The efficiency of this yeast in converting crude glycerol to citric acid is similar to that from glucose [53]. Crude glycerol has also been used as carbon source to obtain vitamin K_2_ [54] and erythritol [55]. 

The main objective of this review is to highlight the use of crude glycerol as starting material for chlorinated intermediates and end products. In particular, we discuss the state-of-the-art in several processes for the synthesis of these compounds, with emphasis on the improvements made in the last two decades. Firstly, the manuscript describes the synthetic methods for chlorinated derivatives of glycerol. The further transformation of chlorinated derivatives in additional downstream products is also described. The more recent contributions of the authors’ research group in the application of chlorinated derivatives of glycerol are also presented. Finally, some future perspectives of these compounds and the evolution of the biodiesel and other related industries are discussed. 

## 2. From Glycerol to Synthetic Intermediates

### 2.1. Synthesis of Chlorohydrins by Glycerol Hydrochlorination

**Figure 2:**
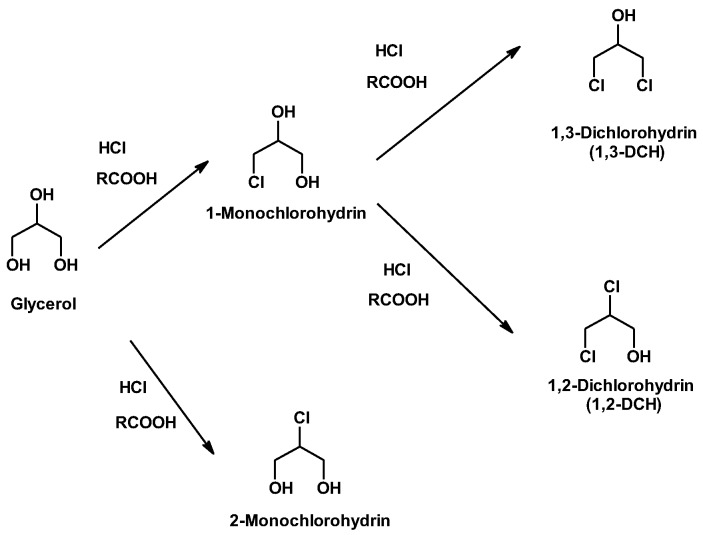
Syntesis of DCH by two-step glycerol hydrochlorination.

An application of glycerol that has attracted significant attention is the production of chlorohydrins [32,34,43,44,51,56,57,58]. Figure 2 shows the synthesized chlorohydrins, using this approach.

Table 1 shows the current approaches to synthesize dichlorohydrins (DCH) by the hydrochlorination of glycerol [12,32,34,43,44,51,59,60,61]. Moreover, some approaches have been described where crude glycerol was used because the purification of glycerol involves high costs and is not economically feasible for small- and medium-size plants [62,63]. Crude glycerol chlorination should represent an economic advantage over the traditional propylene-based process, as the cost of this glycerol is minimal [64,65].

The most prevalent synthetic procedures for glycerol chlorination [66,67] are based on the reaction of glycerol with an aqueous solution of hydrochloric acid [34,56,68,69,70,71,72,73]. The synthetic process has been scaled up to an industrial scale [74,75]. However, this process has a number of disadvantages, such as the loss of the catalyst at high reaction temperatures (due to its low boiling point) and the production of water, which causes an increase in the reaction time and makes it difficult to separate the end products.

This reaction can be carried using glycerol and gaseous HCl. The resulting mixture of isomers has been investigated in great detail [76,77,78]. The reaction is carried out isothermically, allowing the control of side reactions [44].

In the first step, monochlorohydrins (mainly 1-monochlorohydrine, 1-MCH, and small amounts of 2-monochlorohydrine, 2-MCH) are obtained by the nucleophilic substitution of OH by Cl. Moreover, 1-MCH is favored by a kinetic control of the process [79]. In a subsequent hydrochlorination reaction, monochlorohydrins are converted to dichlorohydrins (mainly 1,3-dichlorohydrins, 1,3-DCH, and small amounts of 1,2-dichlorohydrins, 1,2-DCH) (Figure 2). This mixture reach on 1,3-DCH is very interesting in the preparation of epichlorohydrin, as is discussed below. 

These reactions are catalyzed by short carboxylic acids, usually acetic acid. Depending on the HCl concentration, the reaction can lead to the MCH isomers or to the DCH isomers. The ratio between MCH and DCH depends on the reaction conditions. Santacesaria et al. have already reviewed that process and have summarized the studies in terms of catalysts, reaction process, mechanism and kinetics, and reactors and processes used [51].

### 2.2. Synthesis of Epichlorohydrin

Epichlorohydrin (ECH) is a chemical used in the production of synthetic elastomers, sizing agents for the papermaking industry, epoxyresins and plasticizers [34,44,51,80]. Some pheromones, anisomycin, propranolol analogues and β-blockers also have ECH as an intermediate [81,82]. Moreover, enantiopure ECH is an important intermediate for the production of optically active pharmaceuticals, such as atorvastatin and L-carnitine, and the preparation of ferroelectric liquid crystals [83]. 

**Figure 3:**
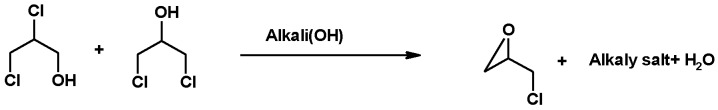
ECH synthesis by alkali treatment of 1,3-DCH.

Industrial methods to synthesize ECH include the use of a mixture of 1,2-DCH (70%) and 1,3-DCH (30%) (Figure 3). This is a disadvantage of the process, since 1,2-DCH is much less reactive than 1,3-DCH [32,33,34,51]. This mixture is currently obtained by propylene chlorination. The alkali treatment of this mixture yielded ECH [44]. ECH can also be obtained by the allyl acetate method. Allyl acetate is hydrolyzed to allyl alcohol, which is chlorinated [44,45]. Both methods are based on the oil industry, since the starting materials are obtained from refinery processes [78]. An additional disadvantage of the process is that the raw materials, such as propylene and chlorine, are flammable and toxic, respectively [61]. These factors have prompted the search for alternative procedures based on sustainable methods and renewable raw materials to synthesize ECH [44]. 

Several chemical and biological approaches [84,85,86] have been suggested as alternatives to prepare ECH [86] from chlorohydrins.

#### 2.2.1. Enzymatically Catalyzed Synthesis of ECH

The intramolecular nucleophile displacement of vicinal halohydrins to the corresponding epoxides can be catalyzed by halohydrin dehalogenases (HHDHs, HheC and EC 4.5.1.X) from microbial origin [87,88,89]. However, a number of studies reported that the biotransformation of 1,3-DCH into ECH by recombinant *Escherichia coli* expressing halohydrin dehalogenase is limited by product inhibition, one of the reasons for the low ECH productivity [90]. Zou et al. proposed a resin-based ISPR biocatalytic process to avoid this inhibition [90]. The method consists in the addition of HZD-9 macroporous resin. HZD-9 improved the overall productivity of the process yielding 88% of ECH (Table 2, entry 2.2) [90]. This high yield demonstrated that this method was an effective way to eliminate product inhibition. Alternatively, halohydrin dehalogenases insensitive to product inhibition have been described [85,91,92]. Thus, *S*-ECH was produced in good enantiomeric excess (92.3% *ee*) and 92% yield, using a HheC mutant (Table 2, entry 2.4) [91]. Improved *ee* (99%) and similar yield (92%) were achieved by using halohydrin dehalogenases (HHDHs) coupled to epoxide hydrolases (EH) (Table 2, entry 2.5) [91]. The production of ECH was also described by using a novel HHDHTm, from *Tistrella mobilis* ZJB1405 (cloned and over-expressed in *E. coli*), with a 75% yield, but with low enantioselectivity compared to other reported HHDHs (Table 2, entry 2.1) [92]. In addition, *HheC* in presence of NO_2_ allowed the synthesis of *R*-ECH with high *ee* (99%) but low yield (41%) (Table 2, entry 2.3) [85]. An alternative method for preparing chiral ECH is the kinetic resolution of its racemate by epoxide hydrolases (EH), which catalyze the opening of the epoxide ring to the corresponding diol in the presence of water [93,94]. It should be noted that HHDH produces mainly *S*-ECH and recombinant EH produces *R*-ECH. As an example, Kim et al. performed the resolution of *R*,*S*-ECH by using recombinant EH, yielding enantiopure (100% *ee*) *R*-ECH (Table 2, entry 2.6) [95]. Lee et al. prepared *R*-ECH with almost similar yield (28.5%) and *ee* (99%) [96]. Jin et al. improved the yield (42.7%) (Table 2, entry 2.8) but reported substrate and product inhibition when the substrate concentration was higher than 320 mM [84]. It should be highlighted that 50% is the highest yield that can be achieved when performing kinetic resolution of a racemate.

#### 2.2.2. Chemical Synthesis of ECH

The chemical synthesis of ECH from dichlorohydrins has been studied extensively. Typically, 1,3-DCH and 1,2-DCH can be transformed into ECH by dehydrochlorination in the presence of alkali hydroxides. Alkaline hydroxides increase the nucleophilicity of OH, which produces the epoxide by substituting one of the chlorines. This reaction is very fast and requires special attention due to the easy occurrence of side reactions.

Various studies (Table 3) have been devoted to the reaction conditions. The composition of the reactive mixtures was studied [97], concluding that 1,2-DCH is much less reactive than 1,3-DCH, although primary alkyl alcohols are more acidic than secondary alkyl alcohols. The influence of the reactor on the reaction kinetics [98,99] and of the cation on the 1,3-DCH dehydrochlorination [59,61,97,100] was also studied.

Lari et al. carried out dehydrochlorination of DCH in the gas phase, in the presence of mixed heterogeneous oxide prepared from hydrotalcite of Al and Mg, which allowed yields of ECH up to 60% [60]. However, this is the lowest yield compared to other chemical processes (Table 3). Alternatively, solid catalysts were prepared by the equivalent-volume impregnation method, using γ-Al_2_O_3_ as a carrier, whereas nitrates and chlorides of the three alkaline earth metals (Mg, Ca and Ba) were employed as precursors. Under optimized conditions, a 90% yield was achieved by using 10BaO/γ-Al_2_O_3_ at 270 °C [101]. Chemical reactions can provide ECH with yields up to 99%, a value slightly higher than the best yield obtained using HHDH+HE [91]. The use of this biotechnological approach allows the synthesis of the S-ECH enantiomer with a 99% *ee*. In addition, the biotechnological approaches avoid the presence of by-products, such as chloroacetone, glycidol, diglycidyl ether and polyglycerols, that are very usual when ECH is synthesized by using the chemical approaches. Moreover, the use of alkaline hydroxides leads to a large amount of salt wastes, thus compromising the sustainability of the technology. Nevertheless, chemical approaches allow working in higher reagent concentration than biotechnological approaches, a usual drawback of the biotechnological approaches from an industrial point of view.

### 2.3. Sinthesis of Dichloropropyl Esters from Glycerol

**Figure 4:**
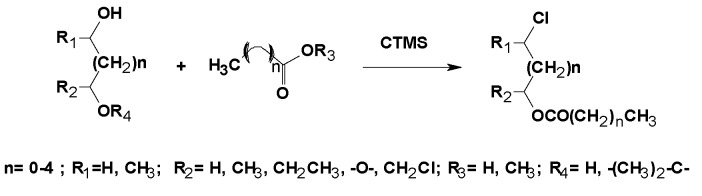
Synthesis of chlorohydrin ester, using carboxyl derivatives, glycerol and CTMS as reagents.

The one-pot synthesis of chlorinated derivatives by using crude glycerol or other polyols as starting materials and chlorotrimethylsilane (CTMS) was described by the authors’ research group (Figure 4) [103]. These chlorinated derivatives showed no effect over fungi and bacteria in preliminary studies, (unpublished results), indicating that these compounds are less toxic than the parent chlorohydrins. Consequently, they could be used instead of 1,3-DCH in some equivalent reactions.

The alkyl chain structure of the carboxylic acid had a clear influence on the regioselectivity of the reaction. Long chains increased the regioselectivity toward the α-chloroalkyl and 1,3 dichloroprop-2-yl radicals, whereas short chains and electron withdrawing substituents on the α carbon reduced regioselectivity [104]. An increase in the degree of substitution of functional groups with α-electron donors led to an increase in the regioselectivity of the reaction [79]. Regioselectivity decreased with increasing temperature, which indicated a kinetic control of the process [105].

**Figure 5:**
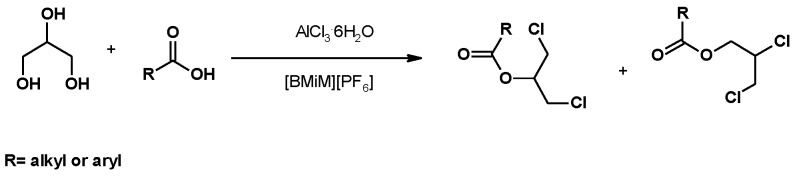
Synthesis of dichloropropyl esters from glycerol and a carboxylic acid, using an ionic liquid.

The synthesis of chlorohydrin esters from glycerol using an ionic liquid as a solvent and hydrated aluminum chloride as a source of chlorine was also described [106]. This approach allowed the use of hydrated aluminum chloride as a chlorine source, avoiding the use of CTMS, a more expensive reagent. Alkyl and aryl acids were used to synthesize chlorohydrin esters, although yields largely depended on the carboxylic acid used. Nevertheless, the corresponding 1,3-dichloro-2-propyl ester was always the main regioisomer (Figure 5). 

## 3. From Building Blocks to End Products

### 3.1. Synthesis of Non-Cyclic Compounds

#### 3.1.1. Synthesis of Allyl Esters

**Figure 6:**
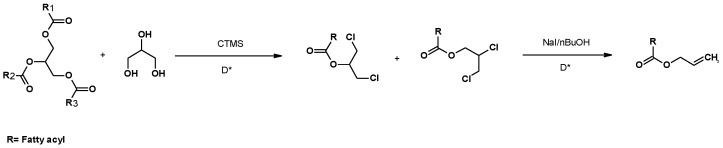
Synthesis of allyl fatty esters, using various fatty materials. Step 1: Conventional heating at 115 °C/48 h and microwave (MW) were 225 °C, 300 W, 17 atm for 3h. Step 2: Conventional heating was 115 °C/48 h, and MW was 150 °C for 25 min.

The synthesis of allyl fatty esters by using various fatty materials (soy oil, frying oils, palm oil, waste animal fats, etc.) and crude glycerol was described by the authors’ research group (Figure 6). Allyl esters were prepared through a two-step reaction, using both conventional and microwave heating [79]. The first step consisted of the synthesis of chlorohydrin esters, as described above. The second step was a Finkelstein-rearrangement–elimination reaction induced by NaI. The reactions were carried out by using butanone [107] or BuOH [79], two solvents that allowed the substitution of a chlorine atom by one iodine atom. Subsequently, the necessary acyl rearrangement and halide elimination took place. The reaction was performed by using conventional or microwave heating. Conventional heating yielded better conversion rates (about 90%, except for olive oil and cocoa industry wastes). Although microwave heating showed a lower conversion rate, and a dark colour was observed in crude products (suggesting degradation), the second step was completed in only 25 min, whereas conventional heating required 48 h [50]. 

Several compounds containing an allyl group are biologically active as insecticides, acaricides and insect repellents [108,109,110]. Allyl fatty acids esters have been suggested as wood preservatives against termites [111]. 

In addition, an ovicidal effect against *Cydia pomonella* (L.) was described for allyl carboxylates [112]. Another application of allyl ester mixtures of higher fatty acids is in polymer synthesis. Highly effective and generally useful copolymers have been prepared from allyl esters [113].

#### 3.1.2. Synthesis of Nitrile Derivatives

**Figure 7:**
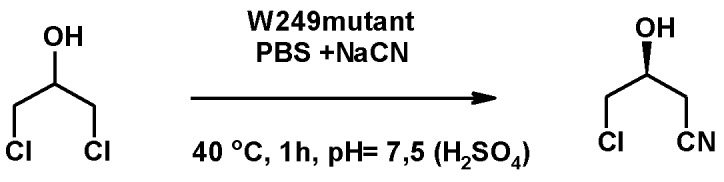
Continuous synthesis of (*S*)-4-chloro-3-hydroxybutanenitrile (*S*-CHBN) from 1,3-DCH and NaCN catalyzed by halohydrin dehalogenase (HheC).

Using halohydrin dehalogenase (HheC) from *Agrobacterium radiobacter*, 1,3-DCH or *R,S*-ECH was used to prepare *S*-4-chloro-3-hydroxybutanenitrile (*S*-CHBN) (Figure 7). The synthesis of *S*-CHBN from *R,S*-ECH yielded a modest enantiomeric excess, whereas the use of 1,3-DCH as substrate led to *S*-CHBN, with 97.3% *ee* after pH optimization. *S*-CHBN was also prepared from 1,3-DCH, with an 86% yield and a 97.5% *ee* in 1 h, using W249F a HheC mutant constructed by site-directed mutagenesis [114]. 

The enantiomer *R*-CHBN was synthesized from 1,3-DCH by using recombinant HheB from *Corynebacterium* sp. N-1074. The final yield was 65%, and the product had an *ee* of 95.2% [115].

Chiral C4 compounds are synthetic units useful for the production of various pharmaceuticals and chiral polymers [116]. As an example, *S*-CHBN is used as a precursor of atorvastatin, a cholesterol-lowering drug [114]. 

#### 3.1.3. Synthesis of Azide Derivatives

##### Synthesis of Diazides

**Figure 8:**
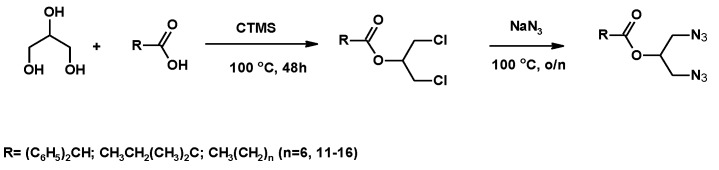
Synthesis of azides from glycerol and carboxylic acids.

To prepare the corresponding diazide derivatives, 1,3-Dichloroprop-2-yl esters were used (Figure 8) [117,118]. The substitution process was carried out by using a conventional methodology to prepare azides [119]. The reaction of 1,3-dichloroprop-2-yl ester with NaN_3_ (Figure 8, See Supplementary Materials Section 1.1.2) yielded 1,3-diazidoprop2-yl esters (70–86% yield), which can be used as crude material for further reactions.

##### Synthesis of Mononoamide Derivatives

**Figure 9:**
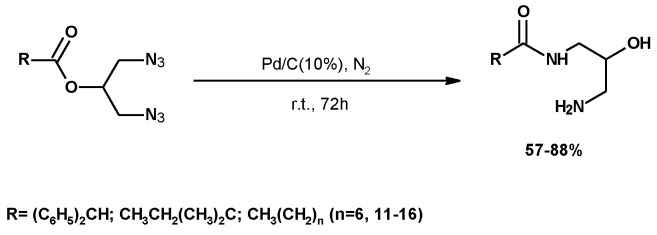
Synthesis of monoamides by hydrogenation of the corresponding diazides.

The synthesis of nine monoamides from crude glycerol and carboxylic acids (C8-C18) was described by the authors’ research group [120]. Diazides were synthesized through the pathway shown in Figure 8. Diazides were reduced by catalytic hydrogenation, under mild conditions, using Pd/C. The reduction resulted in an *O*- to *N*-acyl migration to yield a monoamide (Figure 9). 

The use of monoamides as phase change materials (PCM) in thermal energy storage was investigated. The enthalpy of the monoamides ranged from 25.8 to 149.7 kJ/kg. The highest values of latent heat were of the same order as those of commercial PCMs with low latent heat values, such as paraffin wax (146–210 kJ/kg) [121]. These compounds, which can form at least 4 hydrogen bonds, a powerful assembly tool in terms of PCM activity, were used to demonstrate the effect of hydrogen bonds and alkyl chain on their thermal properties [120].

#### 3.1.4. Sulfonamides

**Figure 10:**
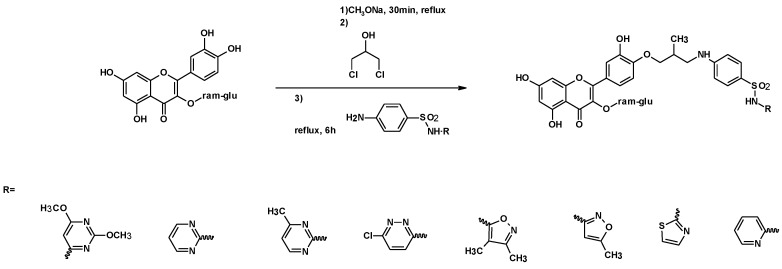
Synthesis of rutine-sulphonamide derivatives, using 1,3-DCH as a linker.

Lupasçu et al. described the synthesis of water-soluble rutin-sulfonamide derivatives with high yields (83–94%). The reaction was carried out by using 1,3-DCH as the linker of rutin and several sulfonamides, resulting in some water-soluble sulfonamide derivatives (Figure 10). The derivatives with pyridine (sulfapyridine) and chloropyridazine (sulfachloropyridazine) showed an equal or even higher antibacterial activity than co-trimoxazole, an antibiotic used to treat a variety of bacterial infections. Co-trimoxazole consists of one part of trimethoprim to five parts of sulfamethoxazole [122].

#### 3.1.5. Synthesis of Polynuclear Metals

**Figure 11:**
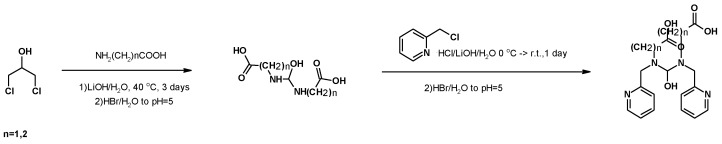
Synthesis of the H_3_hpnbpda ligand.

The synthesis of dinucleating ligands was carried out by using 1,3-DCH [123,124]. The synthesis was a two-step reaction (Figure 11) [124,125]. The symmetrical dinucleating ligand (H_3_hpnbpd) holds two carboxyl groups and two pyridine arms. While Patra et al. synthesized the ligand by reacting glycine with 1,3-DCH in the first step and obtaining the ligand with a 75% yield after a second reaction step with 1-chloromethylpyridine, Haldar et al. used β-alanine instead of glycine as a reagent, achieving the corresponding ligand, with a 73% yield.

**Figure 12:**
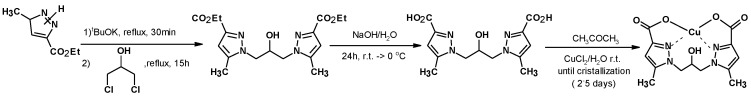
Synthesis of the copper complexes CuL1.

Cooper (II) complexes were also obtained via the synthesis of a bipyrazolic ligand, bearing two carboxyl groups (Figure 12). The first step was similar to the previous one, 1,3-DCH reacted with two molecules of the corresponding pyrazole derivative. The final Cu(II) complex showed good catalytic properties in the oxidation of catechol [126].

Polynuclear metals have many potential applications, e.g., therapeutic agents (e.g., photocleavage of DNA), photovoltaic components, photocatalysts, magnetic materials and tuneable chemical sensors [127,128,129,130]. 

#### 3.1.6. Glycoconjugate Synthesis

**Figure 13:**
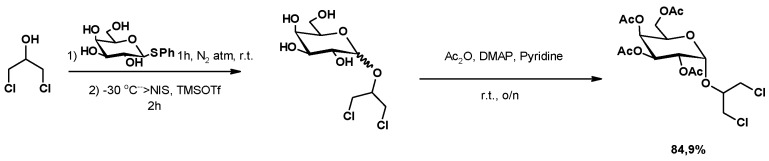
Synthesis of 1,2-*cis*-alkyl tetra-*O*-acetyl glycosides, using 1,3-DCH.

In the synthesis of 1,2-*cis*-alkyl glycosides, 1,3-DCH was used. Figure 13 shows the synthesis of 1,3-dichoroprop-2-yl-2,3,4,6-tetra-*O*-acetyl-α-d-galactopyranoside. The first step consisted of the substitution of the thiophenoxy group of the hemithioacetal of β-d-galactopyranoside by 1,3-DHC. This substitution allowed the synthesis of the corresponding α-d-galactopyranoside alkyl isomer. Finally, the peracetylation of the free alcohol led to the final product, with an 84.9% yield [131]. 

**Figure 14:**
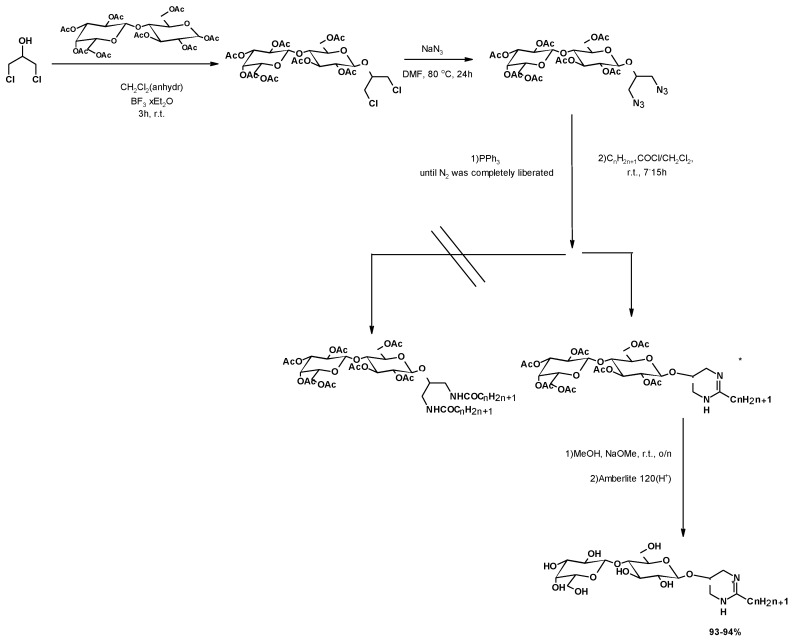
Staudinger reaction of lactose based diazides with fatty acids.

In a similar approach, Salman et al. described the attempt to synthesize diamide-linked bi-antennary surfactants with close structural similarity to natural glycol-glycerolipids [132]. The starting peracetylated disaccharide reacted with 1,3-DCH, in the presence of a Lewis acid (BF_3_), to yield the corresponding alkyl sugar. The substitution of both chlorine atoms by the azide group led to the corresponding diazide. However, the Staudinger-based coupling of fatty acid chlorides did not provide the expected diamide, obtaining the cyclic coupling products instead (Figure 14).

Carbohydrates and glycoconjugates are essential components of the cell membrane. They participate in many functions [133]. Therefore, the chemical synthesis of these glycoconjugates (proteoglycans, glycolipids and glycoproteins) is important for the study of their biological functions. As an exemple, glycol-glycerol lipid amide analogues exhibit very high Krafft temperatures [132]. Anomerically pure alkyl glycosides are used as building blocks to achieve stereoselective synthesis of these structures. Some of them (mostly propargyl and allyl glycosides) [134,135] are also essential components for the construction of microarrays [136,137] and glycodendrimers [138]. 

#### 3.1.7. Funcionalization of Aza-Heterocyclic Compounds

Chlorohydrins and ECH were used to prepare derivatives of pyridine [139,140,141], phtalazines [142,143,144], oxazolidinone [122,145,146], triazinones [147], thioglycoside [148] and aziridines [149]. *N*-Heterocycles have wide applicability as antibiotics [150,151,152]. The evaluation of novel agents for antimicrobial activity is a very important field of study due to the emergence of bacterial resistance to classical antibiotics.

##### Pyridine Derivatives

**Figure 15:**
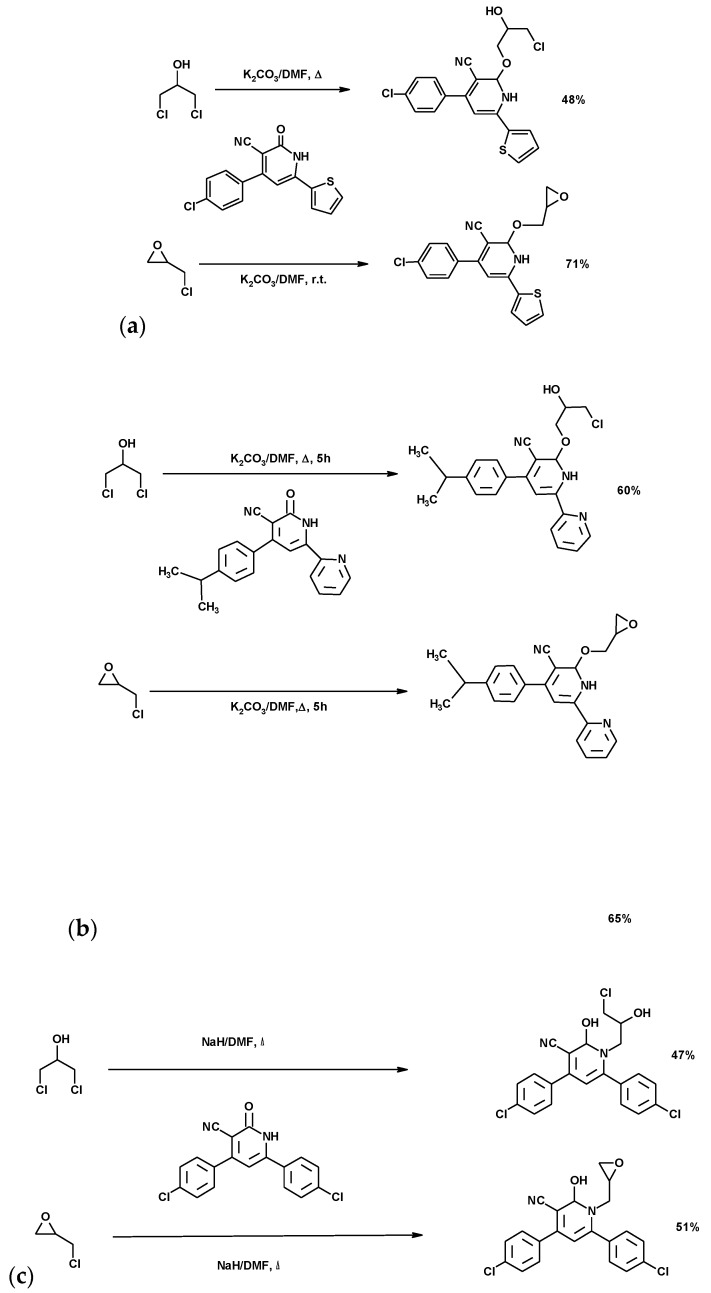
Synthesis of pyridine derivatives (**a**) Moustafa et al. synthesis [139]; (**b**) synthesis described by Saad et al. [140]; (**c**) Shamroukh et al. niconitrile synthesis [141].

The synthesis of *O*-alkyl nicotinonitriles by the reaction of 1,3-DCH or ECH with pyridin- 2(1*H*)-one in presence of K_2_CO_3_ is shown in Figure 15a,b. A similar reaction is described in Figure 15c. The 1,3-DCH or ECH reacted with pyridin-2(1H)-one in the presence of NaH, affording the corresponding N-linked products. K_2_CO_3_ favored the O-alkylation of the lactam, while NaH favored the *N*-alkylation. The derivative synthesized from ECH by Moustafa et al. showed moderate antibacterial activity compared to the standard drug, while the dichloropropanol derivative showed no activity against the tested microorganisms [140]. The compounds synthesized by Saad et al. showed antibacterial effects but no activity against the target fungal strains [140]. However, the compounds synthesized by Shamroukh et al. showed remarkable cytotoxicity activity against MCF-7 and HepG2 cell lines [141]. 

##### Synthesis of Aziridine Derivatives

**Figure 16:**
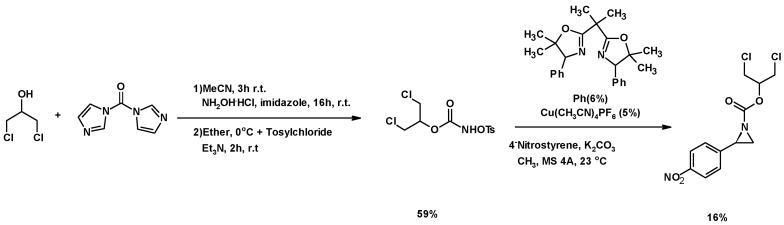
Synthesis of aziridine derivatives using 1,3-DCH.

Lebel et al. described the synthesis of a *N*-tosyloxycarbamate using 1,3-DCH and tosyl chloride. The use of a chiral bis(oxazoline) copper complex with the *N*-tosyloxycarbamate yielded the asymetric aziridines with a enantiomeric *R*/*S* ratio of 4:1 (Figure 16) [149].

Aziridines are the smallest nitrogen-containing heterocycles. Although aziridine moiety is present in few natural products [153], they display important biological activities [154,155]. Aziridines have been introduced into various structures, to create novel chemotherapeutic agents [156,157]. 

In organic chemistry, aziridines are valuable building blocks. As an example, their reaction with many nucleophiles can result in ring-opening reactions [158,159,160,161]. They can also be used as key intermediates in diversity-oriented synthesis of alkaloids [162]. Aziridines have been used in the asymmetric total syntheses of renieramycins M and G and jorumycin, marine bioactive compounds from a blue sponge and a molusc, respectively [163]. Aziridines are masked 1,3-dipoles that react with alkenes, alkynes, nitriles and carbonyl compounds to produce various [3+2] cycloadducts [164]. 

##### Synthesis of 1,2,4-Triazinones Derivatives

**Figure 17:**
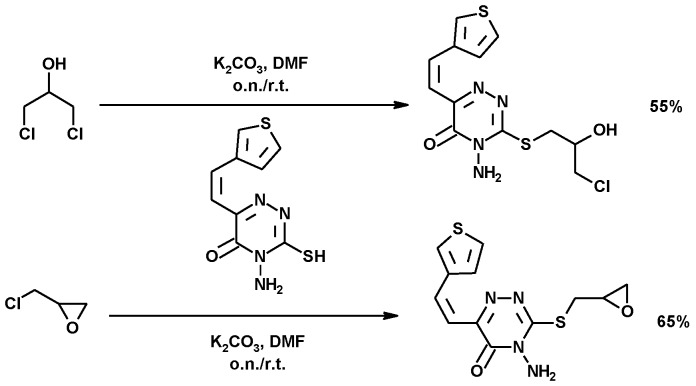
Synthesis of 1,2,4-triazine -thiophene- derivatives.

The synthesis of *S*-alkyl 4-amino-3-mercapto-6-(2-(2-thienyl)vinyl) -1,2,4-triazin -5(4H)-one derivatives, using 1,3-DCH or ECH, is shown in Figure 17. Potassium carbonate in DMF was again used as a base, to improve the nucleophilicity of the S atom, preserving the epoxy group in the final product. These compounds showed moderate anticancer activity [147].

##### Synthesis of Pthalazine Derivatives

**Figure 18:**
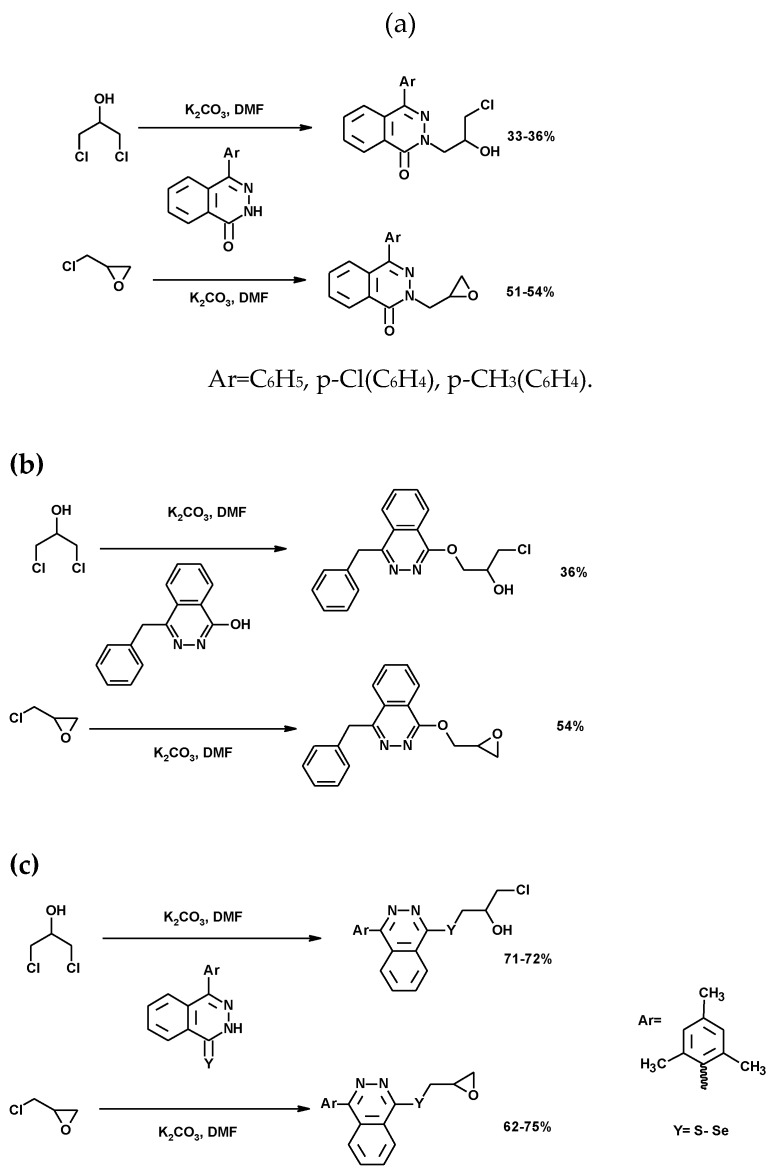
(**a**) Synthesis of *N*-alkylated phtalazines [142]; (**b**) synthesis of 1-oxo alkylated phtalazine [144]; (**c**) synthesis of Se- and *S*-alkyl phthalazines derivatives [143].

The reaction of 1,3-DCH and ECH with arylphthalazinone yielded phthalazin derivatives [142,143,144,147]. Figure 18a shows the *N*-alkyl products resulting from the nucleophilic attack of the nitrogen in the phthalazinone on 1,3-DCH (33% to 36% yield) or ECH (51% to 54% yield). This attack was promoted by the presence of K_2_CO_3_. The loss of aromaticity, when an aryl radical was substituted by a benzyl radical, allowed the regiospecific nucleophilic attack of the oxygen instead of the nitrogen on 1,3-DCH and ECH (Figure 18b) [144]. The reaction of ECH occurred via ring opening–ring closing of the oxirane nucleus (54% yield), while the reaction with 1,3-DCH, was described as a SN_2_ reaction to yield *O*-(3-chloro-2-hydroxypropyl) phthalazine (36% yield). Finally, Se- and *S*-alkyl phthalazines were also synthesized, with yields in the range of 71–72% and 62–75% for the 1,3-DCH and ECH, respectively (Figure 18c). All compounds showed moderate-to-high antimicrobial activity in comparison with standard drugs [142,143]. 

#### 3.1.8. Synthesis of Polymers

**Figure 19:**
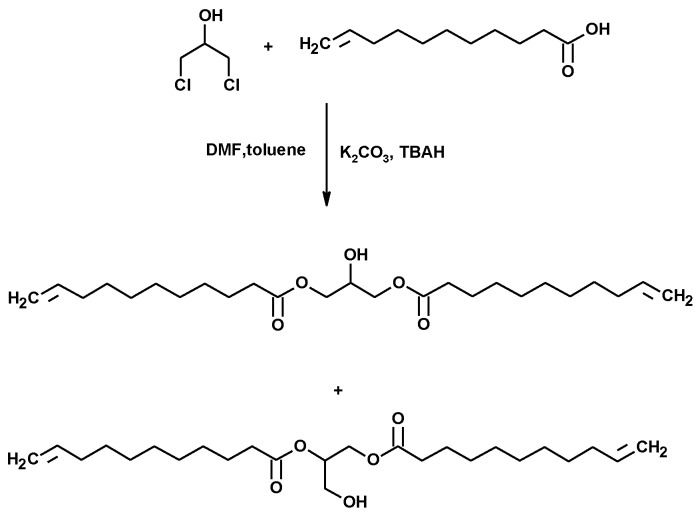
Dimer synthesis of alkenyl fatty acids, using 1,3-DCH as a linker.

**Figure 20:**
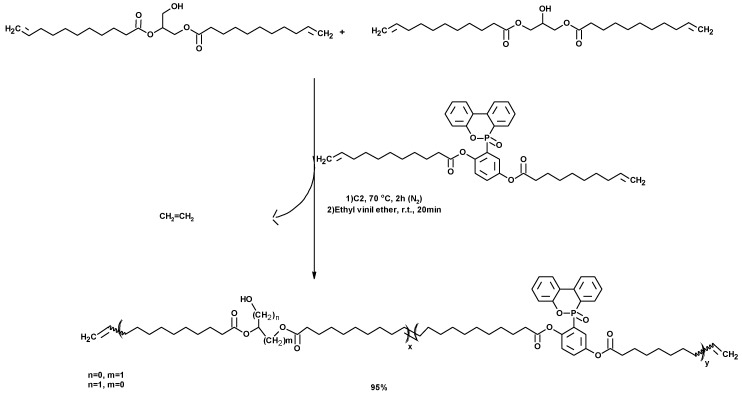
Synthesis of phosphorus-containing polyesters via ADMET copolymerization in presence of Grubbs 2nd generation catalyst (C2).

De Espinosa et al. described a plant-oil-based diene containing hydroxyl groups ten years ago. The diene was prepared by the esterification of ω–alkenyl carboxylic acids (Figure 19) with 1,3-DCH. A phase-transfer catalyst was used due to the high difference of polarity between both reagents. The dimer was polymerized via ADMET polymerization, using a Hoveyda–Grubbs 2nd generation catalyst. It was also copolymerized with an α,ω-diene bearing a DOPO pendant group, also using a Grubbs 2nd generation catalyst (Figure 20). The resulting phosphorus-containing polyesters showed molecular weights up to 7000 Da [165]. The crystallinity of these polyesters decreased as the amount of DOPO-based comonomer (M2) increased. Totally amorphous polymers were obtained for the highest M2 content. Some of these plant-oil-based polymers showed glass transition temperatures ranging from 35 to 52 °C, good thermal stability and relatively good flame retardancy, despite their high aliphatic (fatty acid) content.

Moreover, 1,3-DCH was also used to prepare polymers with specific properties [166]. These polymers are used in many areas, because good flame retardancy for polymeric materials is of great concern to both consumers and manufacturers [167].

### 3.2. Cyclic Compounds

#### 3.2.1. Synthesis of Oxo-Heterocycles

**Figure 21:**
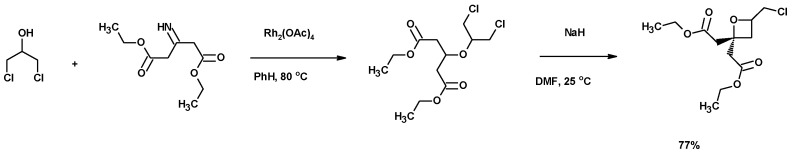
Synthesis of oxetane rings described by Davis et al. [169].

To synthesize oxetane and carbonate compounds, 1,3-DCH and ECH were used, respectively. In addition, 1,3-DCH was used to synthesize a 1,3-dichloroprop-2-yl ether by the catalyzed Rh_2_(OAc)_4-_ substitution of an imino group (Figure 21). The subsequent abstraction of the β-proton of the diester by NaH led to the corresponding chloromethyloxetane with a 77% yield [168]. 

Oxetane is a motif found in natural products and biologically active compounds. Oxetanes are widely used as intermediates in chemical synthesis, such as featuring ring expansion and opening [170,171,172,173,174,175,176,177], rearrangement processes [178,179] or in polymer synthesis [180,181,182,183,184,185,186,187,188]. They are used in drug discovery [189,190,191,192,193,194,195], as they are considered stable adjuncts to adapt solubility, lipophilicity and other physicochemical properties toward drug-like molecules [90,189,190,196,197]. As an example, oxetans show, as a result of their low lipophilicity, a higher metabolic robustness than larger oxygen heterocycles [198,199]. 

**Figure 22:**
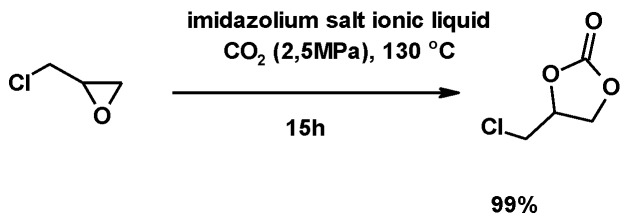
Synthesis of cyclic carbonates, using vinyl-functionalized di-imidazolium salts polymers as the catalyst.

Bobbink et al. described the synthesis of a cyclic carbonate by the cycloaddition of CO_2_ to ECH (Figure 22). The reaction catalyzed by an imidazolium salt led to the selective addition of CO_2_ to the epoxide with a 99% yield—a very high yield, considering the thermodynamic stability of CO_2_. This approach is of particular interest in CO_2_ gas recovery, since cyclic carbonates may be used in polymer synthesis [200]. 

#### 3.2.2. Synthesis of Aza-Heterocycles

Moreover, 1,3-DCH and ECH were also used to prepare oxazolidinones and triazoles.

##### Synthesis of Oxazolidinones

**Figure 23:**
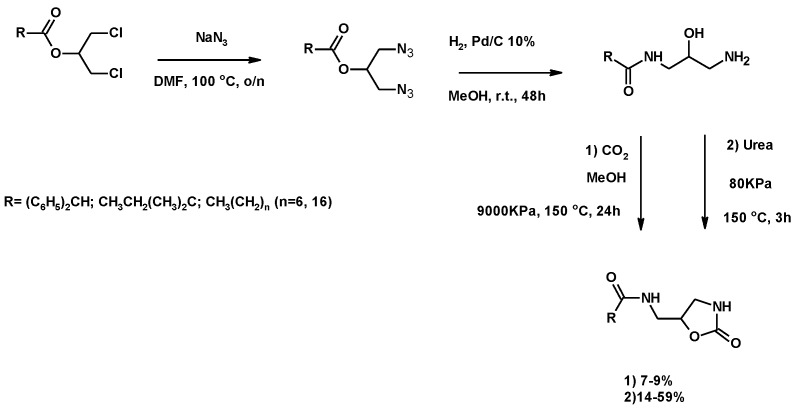
Synthesis of oxazolidinones from dicholoralcohol esters.

The synthesis of oxazolidinones was described by using 1,3-DCH [145,146], or 1,3-dichloropropan-2-yl esters [118]. Figure 23 shows the synthesis of oxozalidinones, starting from 1,3-dichloropropan-2-yl esters described by the authors’ research group [118]. 

The first step was the preparation of the diazide as previously described (Section 3.1.3; Figure 8). The hydrogenation of the azide derivatives led to the corresponding monoamide by a *O*- to *N*-acyl migration (Figure 23) [120]. The monoamide reacted with urea or CO_2_, under the conditions shown in Figure 23, (see Supplementary Materials Section 1.2.2) to achieve the corresponding oxazolidinone. The final yield, determined by ^1^H RMN, for the urea reaction ranged from 14% to 59%. When the reaction was carried out with CO_2_ at a high pressure and temperature, the corresponding urethane was obtained with a 7% to 9% yield. Unfortunately, these urethanes showed low stability when purified [118].

**Figure 24:**
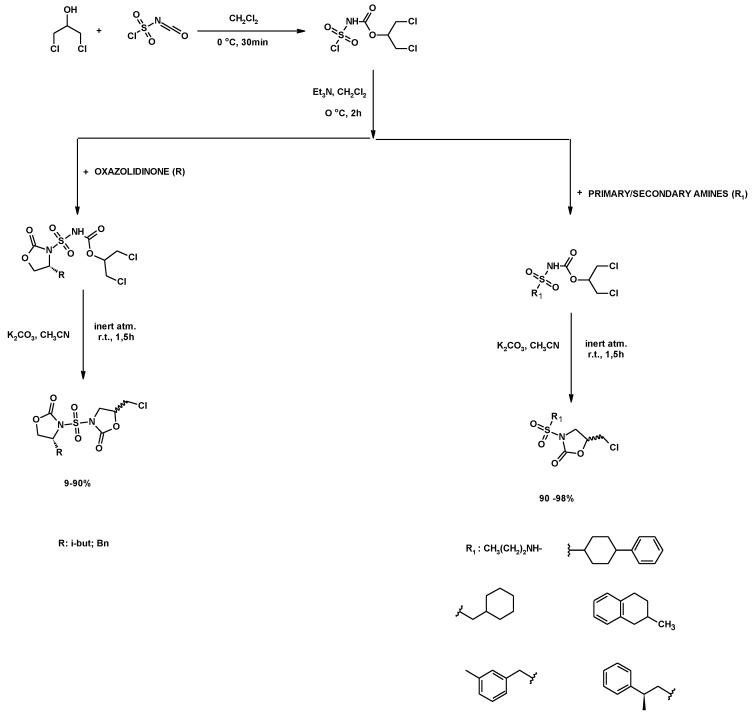
Synthesis of sulphamoyloxazolidinones from 1,3-DCH.

Both 1,3-DCH and chlorosulphonyl isocyanate were the starting materials to obtain a carbamate which was used afterward to synthesize sulfonamides bearing oxazolidinone rings [145,146]. The resulting carbamate reacted with oxazolidinones yielding *N*-oxazolinone sulfonamide. Finally, the addition of a base (K_2_CO_3_) allowed the reaction of the NH in the sulfonamide with one of the carbons bonded to a chlorine atom, yielding the corresponding *N*,*N*-bis-oxazolidinones-sulfones. The compound with an isobutyl radical was synthesized with a 90% yield, while the benzyl substituted compound was synthesized with only a 9% yield (Figure 24, R). Alternatively, the reaction between the carbamate and amines led to substituted sulfamides. Finally, the carboxylsulfamides in presence of potassium carbonate in acetonitrile led to 5-chloromethyl-2-oxazolidinone sulphonamides with a chiral center at the 5-position (90% to 98% yield) (Figure 24, R_1_) [145,146]. The antibacterial activity of these compounds was evaluated. Most of the compounds showed moderate-to-good antibacterial activity [145]. 

##### Synthesis of Triazole Derivatives

**Figure 25:**
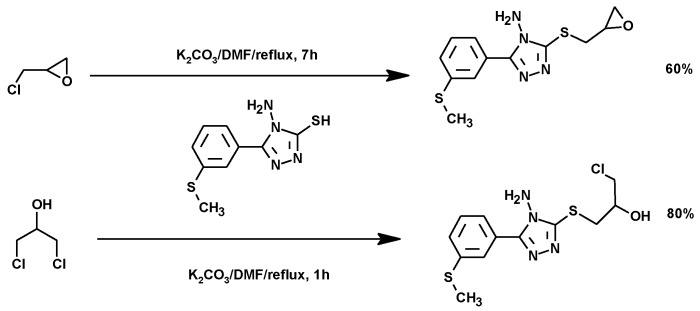
Synthesis of triazole-thioglycoside from ECH and DCH.

Triazoles are important molecules for chemical synthesis and also as bioactive molecules. Figure 25 shows the synthesis of an *S*-acyclonucleoside by alkylation of 5-(2-methylthio)phenyl-1,2,4-triazole-3-thiol with 1,3-DCH or ECH. The 1,2,4-triazole thioglycoside was obtained by using potassium carbonate in DMF. Potassium carbonate was again used as a base to improve the nucleophilicity of the S atom preserving the epoxy group in the final product [148].

Triazole acyclic nucleosides synthesized from ECH have moderate-to-high antifungal and antibacterial activities compared to standard drugs [148]. Another application of triazoles is the preparation of microliter plates. Microliter plates were coated with hydrocarbon chains bearing a sugar moiety. This sugar motif was attached to the alkane by a 1,3-dipolar cycloaddition. These coated plates were used to develop new microfabrication methods for application in the screening of bioactive carbohydrates and enzymatic activities [201]. Based on this idea, the synthesis of novel compounds with alkyl chains bearing two sugar moieties per chain in their head was designed. The synthesis was carried out by using diazide derivatives and alkinyl glycosides, which were prepared by using the Fischer glycosilation reaction [23]. The corresponding triazole derivatives were synthesized by the 1,4-regioselectivity copper(I)-catalyzed azide-alkyne cycloaddition reaction (CuAAC). This approach allowed the synthesis of 1,4-disubstituted 1,2,3-triazoles as unique regioisomers [202]. Yields ranged from 40% to 57%, after column purification (see Supplementary Materials Section 2.2.2). 

Microliter plates bearing the synthesized compounds were prepared. The interactions between the alkane sugars and C-lectin glycoproteins were measured by using surface plasmon resonance spectroscopy (SPR) in a high-throughput multichannel mode with a GLC chip. However, no response was achieved on SPR sensograms, even at the higher concentration (100 μM solution).

**Figure 26:**
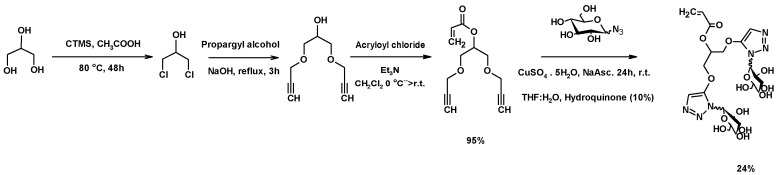
Synthesis of bis-triazol monomers from glycerol.

Polymers bearing one sugar moiety per chain act as competitors for gp120, an epitope of AIDS, to interact with DC-SIGN [203]. Considering this, the authors’ research group planned the synthesis of polymeric structures similar to those already described [203] but with two sugar moieties per chain and using glycerol as a starting material. Figure 26 shows the synthetic strategy used to prepare the corresponding monomer. Then, 1,3-DCH was prepared from crude glycerol, using chlorotrimethylsilane and acetic acid as the catalyst [105]. The reaction of 1,3-DCH with propargyl alcohol in basic media afforded 1,3-bis(prop-2-yn-1-yloxy)propan-2-ol. The basic media increased the nucleophilicity of the hydroxyl in propargyl alcohol, which is more acidic than the secondary alcohol of 1,3-DCH. The esterification of 1,3-dialkynyloxy-2-propanol with acryloyl chloride yielded 1,3-bis(prop-2-yn-1-yloxyl)propan-2-yl prop-2-enoate (see Supplementary Materials 2.2.5) [118]. 

Finally, the reaction of 1,3-bis(prop-2-yn-1-yloxyl)propan-2-yl prop-2-enoate with a sugar azide led to the desired monomer through a CuAAC reaction. This last step was performed in H_2_O:THF (1:1), with a 10% hydroquinone as a polymerization inhibitor. 

**Figure 27:**
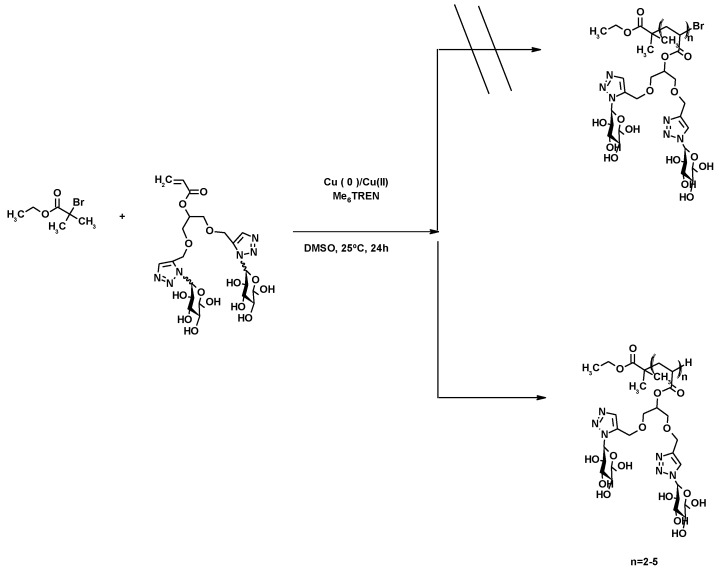
Proposed structure for the dead polymer synthesized through SET-LRP polymerization.

The polymerization of the glycomonomer D-mannose was intended, using Cu(0)/Cu(II)/Me_6_TREN as a catalyst with EBiB as initiator (Figure 27). Although the expected polymer with the terminal bromine was not detected through using the MALDI-ToF technique, dead polymer chains (with terminal hydrogen) and two-to-five added chains were obtained. The exchange of the bromine by the proton was mainly caused by disproportionation and chain transfer side reaction, which led to the loss of the terminal bromine (see Supplementary Materials Section 2.2.6) [118]. 

**Figure 28:**
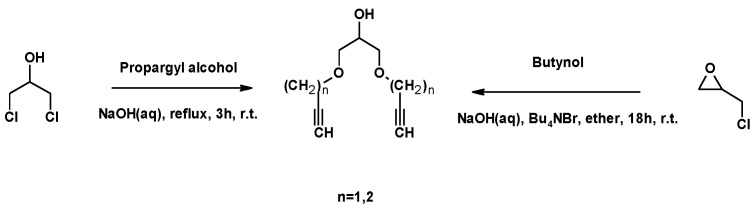
Synthesis of glycerol-type linking arms based on alkenyl motifs using DCH and ECH.

A similar starting approach was described by Legros et al. for the synthesis of novel β-cyclodextrin dimers. Glycerol-type linking arms were synthesized from 1,3-DCH or ECH, using NaOH as a basic catalyst. Propargyl alcohol and butynol were used as nucleophilic reagents. A phase transfer catalysis (Bu_4_NBr) was also used in the reaction between ECH and butynol (Figure 28) [118].

**Figure 29:**
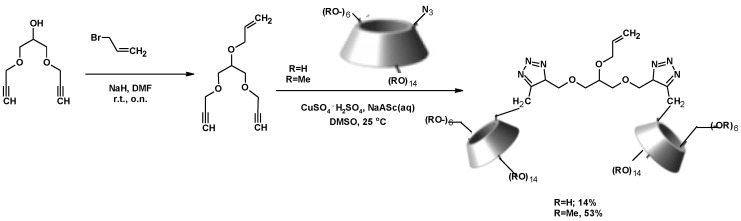
Synthesis of β-CD dimers with a functionalized glycerol linker.

These glycerol-type linkers were used to synthesize β-CD dimers by a CuAAC reaction (Figure 29) [204,205,206,207]. One of these CD dimers showed unusual conformations in aqueous solutions. These conformations depended on the length of the linking arm between the two cyclodextrins [208,209]. 

Due to their unique cup-like structures, CDs are known to form inclusion complexes in aqueous solution. CDs have a wide range of applications that include the areas of drug delivery [210,211], analytical chemistry [212], artificial enzymes [213], photochemical sensors [214], food technology [215], catalysis [216] and nanostructured functional materials [217]. In comparison with CD monomers, bridged bis(β-CD) derivatives allow two hydrophobic cavities to be in close vicinity, thus improving the desired properties. Moreover, the presence of functional linkers between the two CDs can supply a well-organized pseudo-cavity that may afford supplementary binding properties [218,219].

#### 3.2.3. Synthesis of Ionic Compounds Based on Quaternary Bis-Ammonium Salts

**Figure 30:**
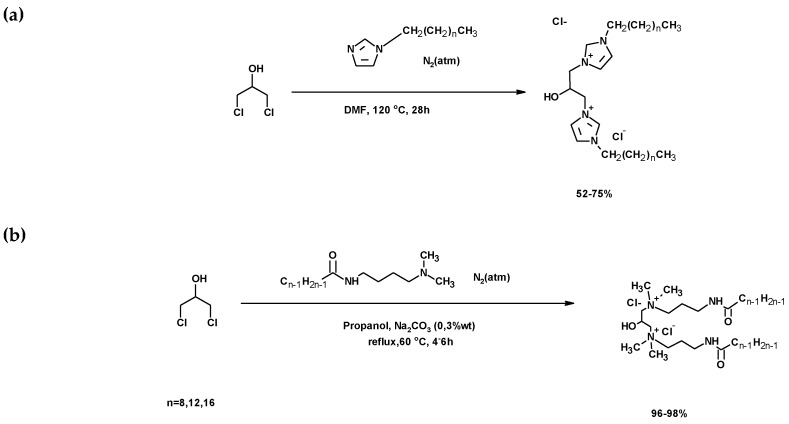
(**a**) Synthesis of gemini imidazolium salts using 1,3-DCH. (**b**) Synthesis of lineal amide-based gemini cationic surfactants using 1,3-DCH.

In the study, 1,3-DCH was used to synthesize gemini imidazolium salts, with an hydroxyl in the spacer group and lateral chains of different length (Figure 30a) [220]. A similar reaction with amines instead of imidazole was described by Song et al., who synthesized bis-quaternary ammonium salt (BQAS) with a hydroxyl in the spacer group [221]. This salt was synthesized by the reaction of 1,3-DCH with *N*,*N* dimethyldodecylamine [221], achieving a 90% yield. BQAS exhibited broad-spectrum bactericidal activity [221]. Another BQAS was synthesized by using monoamides of α,ω-diamines (Figure 30b). All of these syntheses are based on the nucleophylic attack of a tertiary amine to the carbons of 1,3-DCH supporting the chlorine atoms. The presence of a hydroxyl in the spacer group confers tuneable properties to these compounds [222,223].

Imidazolium derivatives showed higher thermal stability than conventional quaternary ammonium gemini surfactants and two-phase transitions before decomposition [220]. Amide-based gemini cationic surfactants presented superior surface/interfacial activities and easy biodegradables, suggesting them as potential products in industrial fields, such as surfactant flooding [224]. 

Gemini compounds have high surface activity and low critical micelle concentration (CMC). These properties enhance their water-solubility and confer a better viscosity than single-chain surfactants at equal molar mass concentration [116,225,226,227,228,229,230,231]. Consequently, their efficiency is improved [232], allowing them to be used in smaller quantities compared to conventional surfactants [232]. These properties enable their industrial use in various fields, such as antiseptics, printing and dyeing, corrosion inhibition, improved oil recovery and synthesis of inorganic materials [224,233,234,235,236]. They can also be used in electro-decoating, stabilization of adhesive polymers, anti-friction agents, mining, paper-making, cosmetics and, more recently, in drug design and delivery [237,238]. Most of them also show strong antibacterial and antifungal activities, becoming safety weapons [230,235,239,240,241]. Their mechanism of action is based on the amphiphilic nature of the gemini group, which allows them to interact with the cell membrane of the microorganisms, causing them to lose their permeability [242].

Gemini compounds are also used as ionic liquids (DDIL). Ionic liquids (ILs) are characterized by unique properties, such as non-volatility, low flammability, tuneable hydrophobicity, environmentally friendly nature, easy recoverability and recyclability [243]. They are of recognized interest for a wide range of applications, such as for solvents, in chemical and enzymatic catalysis [244,245,246], in carbon dioxide capture and separation, in hydrogen generation, in converting thermal energy into electrical energy, for electrochemical energy storage and for converting electrical energy into mechanical energy [247]. It is well-known that the physical and chemical properties of an IL can be tailored by varying the structure of constituent cations and anions [248,249]. Dicationic ionic liquids (DDIL) contain two head groups, linked by a rigid or flexible spacer [250]. This type of IL demonstrates unique characteristics not found in monocationic ILs and other traditional solvents [251]. Moreover, the change in the length of the spacer and the incorporation of functional groups such as thiol, ether, hydroxyl and amino groups in the cations allows tailoring the physical properties of DDIL for specific applications [252]. The DDIL PEG based [253] have also been used as a powerful catalysts for various synthetic transformations [254,255].

**Figure 31:**
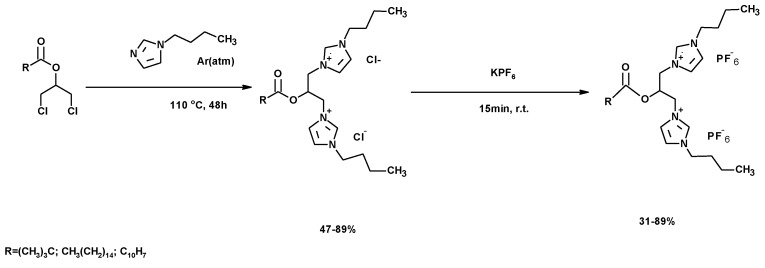
Synthesis of ionic compounds from chlorohydrin esters.

Moreover, ionic liquids (IL) have recently been proposed for thermal storage applications [237]. ILs have thermophysical and chemical properties that may be suitable to be used as heat transfer fluid (HTF) in power plants, using parabolic trough solar collectors, as stated by Van Valkenburg et al. [238]. The authors’ research group described the use of crude glycerol, *N*-butylimidazole and carboxylic acids [256,257] to synthesize diimidazol-1-ium esters, DDILs, with high capacity for energy storage (Figure 31). A counter ion swap was also achieved with KPF_6_, as shown in Figure 31.

The final yields were highly dependent on the carboxylic acid used. The set of bis-imidazolium ester chlorides showed interesting energy-storage properties, as indicated above. However, the substitution of chloride ions by hexafluorophosphate ions yielded a set of compounds with lower PCM capability [256].

## 4. Future Perspectives

It is clear from the above studies that finding cost-effective alternatives to the use of crude glycerol is an active field of research. The synthesis of DCH and dichloropropyl esters is possible from crude glycerol, which implies that ECH and other derivatives can also be synthesized from crude glycerol. Moreover, the synthesis of ECH is faster from 1,3-DCH, the main chlorohydrin isomer synthesized from glycerol, than from 1,2-DCH, the main isomer resulting from propene chlorination. Nevertheless, although three companies (Dow Chemicals, Solvay EPICEROLTechnology and CONSER SpA ECH-EF = Eco Friendly) have developed their own process for producing dichlorohydrins from glycerol and HCl, further work is necessary to identify the most-reliable catalytic mechanism and the best catalyst [51]. Dichloropropyl esters may be a less toxic substitute of 1,3-DCH for some synthesis. However, the current processes to prepare these esters with high yield need expensive reagents (CTMS) or solvents (IL). Further work is necessary to identify cost-effective synthesis for these esters. Crude glycerol can also be used as a carbon source in fermentative processes, although intensive research is still necessary to improve the use of crude glycerol in most of the fermentative processes currently used.

Products synthesized from chlorinated glycerol derivatives have applications in areas such as agriculture, chemistry, healthcare and materials (Table 4). Electrophilic and nucleophilic reagents, as well as some compounds with catalytic and photocatalytic properties, have also been prepared from these chlorinated compounds. Antimicrobial and anticancerinogenic compounds are the main targets for the compounds prepared to be used in medicine, although antiviral, antihypertensive, diuretic and hypoglycemic properties are also present in some of the synthesized compounds. Finally, polymers with different properties, surfactants, ionic solvents and phase-change materials are the main targets in the field of materials.

Despite recognized advances in this field, only a few of the synthesized compounds are already commercial products, while many others are still at the research stage. Further work is therefore needed to synthesize novel compounds with improved properties and to demonstrate the actual application of those compounds still at the research stages. The authors’ research group has also recently demonstrated that crude glycerol can be used to prepare novel deep eutectic solvents (DES), similar to those based on choline chloride. Chloline chloride is substituted by a quaternary ammonium salt synthesized from 1-MCH [258]. This also opens up new opportunities for adding value to crude glycerol.

These studies should also consider alternative approaches under study to prepare biofuels from vegetable oils and fats, avoiding glycerol generation. Gliperol, DMC-Biod or Ecodiesel, likewise, another renewable diesel fuel, known as “green diesel”, are produced by treatment of vegetable oils (cracking, pyrolysis, hydrodeoxygenation and hydrotreating). Other strategies aim to reduce the high viscosity of vegetable oils by mixing them with low-viscosity solvents, in the right proportions, to obtain suitable fuels. In this way, the costs associated with the transformation of vegetable oils and fats can be reduced. Efforts are also devoted to the purification of crude glycerol, although the current processes are still considered too expensive for the actual industrial application, at least for small biodiesel producers [259]. Nevertheless, crude glycerol is also a by-product of the biolubricants industries, one of the top 20 innovative bio-based products described in a recent EU study [260]. Consequently, the production of crude glycerol seems to be a reality for a long time forward.

## 5. Conclusions

Although pure glycerol is currently used in a wide variety of applications, primarily in he cosmetic, food and pharmaceutical industries, the purity of glycerol resulting from the biodiesel industry is far from meeting the purity needed for these applications. This glycerol, also currently known as glycerine (the former name of glycerol), can directly be used as an energy source and as a starting material in chemical synthesis. This latter approach seems more profitable than simply burning glycerol as waste. It has already been demonstrated that chlorohydrins and chlorohydrin esters can be prepared from crude glycerol. Luckily, glycerol is a polyol with many potential applications. Hence, it is easy to substitute some of its hydroxyl groups to obtain chlorohydrins. Chlorine atoms can afterward be substituted by other nucleophiles, resulting in ECH. This intermolecular substitution is favored from 1,3-DCH, the main regioisomer obtained from glycerol. Intramolecular substitutions lead to a large number of intermediate and end products from single molecules to large polymers with applications in agriculture, chemistry, medicine and materials. Among them, the preparation of gemini ionic compounds seems to be one of the more promising areas, considering their properties. Novel DES can be also prepared starting from chlorohydrins. In fact, the more options there are for the applications of glycerol, the more likely it is that biodiesel and biolubricants will become real alternatives for fuel and lubricants in the future. Consequently, interest in developing novel value-added uses for glycerol is increasing.

## Figures and Tables

**Table 1:** Effect of several conditions on the synthesis of DCH.

Reagents	Catalyst	P (atm)/T (°C)	Procedure	Reaction Period	1,3-DCH (Yield %)	Comments	Ref.
HCl(g) +wet glycerol (9%)	Acetic acid (5%)	7.5/110	Batch (glycerol) Continuous (HCl)	4	93% DCH (46:1) (1,3-DCH:2,3-DCH)	HCl pressure has a great effect on glycerol consumption rate and product distribution.	[44]
HCl(g) +glycerol	Acetic acid (0–50%)	0.25–1/105	Semibatch	3	N.P.	Non-catalytic hydrochlorination is a major inconvenient at high temperatures...	[43]
HCl(g) +glycerol	Propionic acid 8%	1/100	Batch(glycerol) Continuous (HCl)	3	41%	No correlation between the acidity strength of the catalyst and the reaction activity was demonstrated.	[32]
HCl (g) +glycerol	Hexanoic acid (5%)	7.5/110	Semibacth	3	N.P.		[61]
HCl(g) +glycerol	Carboxylic acid studied	N.P.	Batch(glycerol) Continuous (HCl)	N.P.	N.P.	Correlation between catalyst p*K*a value and its selectivity toward mono- (p*K*a < 3) or dichlorinated (p*K*a > 4) compounds was found.	[34]

N.P., not provided; DCH, dichlorohydrins; MCH, monochlorohydrins.

**Table 2:** ECH synthesis using biotechnological approaches.

Entry	Enzyme Type	Enzyme from/Mutant	Isomer	*ee* (%)	Yield (%)	Comments	Ref.
2.1	HHDH	*Tistrella mobilis* ZJB1405 (*E. coli*)	S-ECH	N.P.	75	Alkaline pH, 45 °C	[92]
2.2	HHDH	*E.coli* BL21(DE3)	ECH	N.P.	88.3	HZD-9 resin at 10% (*w*/*v*)	[90]
2.3	HHDH	*Agrobacterium radiobacter*	R-ECH	99	41	NO_2_, pH5, 37 °C, 18 min	[85]
2.4	HHDH	P175S/W249P	S-ECH	92.3	93.2.	pH = 10	[91]
2.5	HHDH + EH	N.P.	S-ECH	99	91.2	Enzyme combination	[91]
2.6	EH	*Pichia pastoris* harboring the *Rhodotorula glutinis* EH	R-ECH	100	26.4		[95]
2.7	EH	N.P.	R-ECH	99	28.5		[96]
2.8	EH	*A. radiobacter*	R-ECH	≥99	42.7	Subtract and product inhibition	[84]

N.P., not provided.

**Table 3:** ECH synthesis using basic catalysts.

Reagent	Catalyst	Reactor System	Temperature (°C)	Yield %	Ref.
1,3-DCH	NaOH	Continuous millireactor	30–70	50–99	[59]
1,3-DCH:1,2-DCH(98:2)	Ca(OH)_2_:CaCO_3_:H_2_O (96:4:1, *w*/*w*%)	Pre-reactor/reactor Stripping column	51/64	85–90	[98,102]
1,3-DCH: 1,2-DCH	NaOH	Microreactor	50–80	92	[97]
1,3-DCH	Ba, Ca and Ba/γ-Al_2_O_3__2_	Fixed-bed reactor	150–300	10–90	[101]
1,3-DCH:1,2-DCH Aqueous (5–10 wt%)	Heterogeneous hydrotalcite	Continuous-flow fixed-bed	200	60	[60]

N.P., not provided.

**Table 4:** Summary of the properties of the different products obtained from glycerol based on chloroderivatives.

Field of Application	Property	Current Status	Chemical Compounds	Starting Materials	Section
Agricul-ture	Pesticide	Research	Allyl esters	Chorohydrin esters	3.1.1
	Antimicrobial	Commercial product	1,2,4-Triazinones	DCH/ECH	3.1.7
Chemis-try	Reagent	Commercial product	DCH	Glycerol	2.1
	Reagent	Commercial product	ECH/(*S*)-CHBN	DCH	2.2/3.1.2
	Reagent	Research	Chlorohydrin esters	Glycerol	2.3
	Reagent	Research	Diazides/Monoamides	Chorohydrin esters	3.1.3
	Reagent	Research	Alkyl glycosides/Azidirines/Oxetanes	DCH	3.1.6/3.1.7/3.1.8
	Reagent	Commercial product	Cyclic carbonates	ECH	3.1.8
	Reagent	Research	Oxazolidinones	DCH/Chorohydrin esters	3.2.2
	Analytic sensors	Research	Polynuclear metals /Alkyl glycosides	DCH	3.1.5/3.1.6
	Analytic sensors	Research	Triazoles	DCH/ECH	3.2.2
	Catalyst	Research	Polynuclear metals	DCH	3.1.5
Health	Anti-microbial	Commercial product	Sulfonamides	DCH	3.1.4
	Anti-microbial	Research	Pyridine derivatives	DCH/ECH	3.1.7
	Anti-microbial	Research	Azidirines/Phthalazines/Oxazolidinones/gemini imidazolium salts	DCH	3.1.7/3.2.2/3.2.3
	Anticancer	Research	Azidirines	DCH	3.1.7
	Anticancer	Research	Pyridine derivatives	DCH/ECH	3.1.7
	Antiviral		Sulfonamides/Polynuclear metals	DCH	3.1.4/3.1.5
	Anti-hyper-tensive		Sulfonamides	DCH	3.1.4
	Diuretic		Sulfonamides	DCH	3.1.4
	Hypo-glycemic		Sulfonamides	DCH	3.1.4
Materials	Polymers	Research	Allyl esters	Chorohydrin esters	3.1.1
	Polymers	Research	Polyesters	DCH	3.1.8
	Flame retar-dants	Research	Polyesters	DCH	3.1.8
	Surfactants	Research	Gemini imidazolium and ammonium salts	DCH	3.2.3
	Ionic Solvents	Research	Gemini imidazolium and ammonium salts	DCH	3.2.3
	PCM	Research	Monoamides/gemini imidazolium and ammonium salts	Chorohydrin esters/DCH	3.1.3/3.2.3
	Magnetic materials	Research	Polynuclear metals	DCH	3.1.5
	Photo-voltaic component	Research	Polynuclear metals	DCH	3.1.5

## Supplementary Materials

The following are available online, Oxazolidinones, materials and methods pages S1–S3; Triazole derivatives synthesis, materials and methods (synthetic process) pages S6–S10.
