# Supplementary material for: Preparation and Uses of Chlorinated Glycerol Derivatives
Source: Molecules. 2020 May 28;25(11):2511. doi: 10.3390/molecules25112511 (PMC7321119; doi:10.3390/molecules25112511)
Supplement: Supplementary file 1 [file molecules-25-02511-s001.pdf]

SUPPLEMENTARY MATERIAL

Preparation and uses of chlorinated glycerol derivatives

Anna Canela-Xandri, Mercè Balcells, Gemma Villorbina, Paul Christou, Ramon Canela-Garayoa

1.-SYNTHESIS OF MONOAMIDES AND OXAZOLIDINONES

1.1 REAGENTS AND EQUIPMENT.

2,2-Dimethylbutyric acid (96% Fluka), diphenylacetic acid (99% Acros organics), caprylic acid (>98% Fluka), stearic acid (97% Probus), glycerol (99.5% Fluka), CTMS (ACROS, 98%), sodium azide (99%, Aldrich), Amano-lipase PS-LM (Immobilized on diatomaceous earth, Sigma), Lipozyme IM from *Mucor miehei* (Fluka), Lipase acrylic resin from *Candida antarctica* (Sigma), Amano lipase PS from *Pseudomonas cepacia* (30.000 µ/g Aldrich), *R. oryzae* (produced in the own laboraroty), Baker's yeast (commercial), $FeSO_4{\cdot}7H_2O$ (99%,Probalo), Zn (Probus), NH_4Cl (99.5% Panreac), $(PPh_3)_3RuCl_2$ (98% Strem chemicals), triphenylphosphine (97% Fluka), Pd/C immobilized on charcoal (10% Aldrich), urea (Panreac), 1,3-dioxolan-2-one (98%, Aldrich) Novozyme 435 (Novozymes) were used as reagents and catalysts. Solvents were purchased from Sigma-Aldrich, Aldrich, Fluka, Supelco and Across and were used without further purification. Diazides (**1a**-**1d**) were synthesised from glycerol and the corresponding carboxylic acid according the procedure described by our research group[36]. The crude reaction mixture was not purified, due to its instability, and directly used in further reactions. Purification of the final resulting solid compounds was performed by crystallization.

^{1}H and ^{13}C NMR spectra were recorded on Varian AS400 MERCURYplus (^{1}H, 400MHz and ^{13}C, 100 MHz), using $CDOD_3$, $CDCl_3$ and D_6MSO as solvent. The spectra were recorded at 30°C or 55°C depending on the product solubility and 20 s of relaxing time. Chemical shifts (δ) were reported in ppm relative to the solvent used. Spin multiplicities were reported as

a singlet (s), doublet (d), or triplet (t) with coupling constants (*J*) given in Hz, or multiplet (m).

The melting points were measured by open capillary tubes in Gallenkamp equipment. They are uncorrected.

IR spectra were recorded on a Jasco FT/IR-6300 equipment, in a range between 400 to 4000 cm^{-1}. The equipment is set to take 60 spectra/second with a resolution of 16 cm^{-1} with ATR.

High resolution mass spectra were recorded by direct infusion in a Agilent G6510AA Q-TOF mass spectrometer using ESI ionization.

1.2 METHODS

1.2.1 General procedure for the synthesis of monoamides(Figure 9).

Pd/C Hydrogenation.

In a typical experiment, a suspended mixture of 10% Pd/C (10 wt%) in MeOH (1.0 mL). was added to a stirred solution of the diazide (**1a**-**1d**) (1.0 mmol) in MeOH (1 mL). The system was sealed with a septum. After two vacuum/H_2 cycles, the mixture was vigorously stirred at 25°C under ordinary hydrogen pressure (balloon) for 72 h. The reaction mixture was filtrated through Celite. The solution was concentrated under vacuum to yield white powders for **2b**-**2d** products. Compound **2a** was a colourless oil. The solid crudes were further purified by crystallization and analysed by ^{1}H NMR.

1.2.2.General procedure for the synthesis of oxazolidinone.(Figure 13)

1) Method using CO_2.

In a typical experiment, a solution of monoamides (0.15 mmol) was introduced into the 25 mL steel reaction vessel. Catalyst was added if convenient. The reactor was flushed three times with CO_2 and finally CO_2 was introduced up to the gas bottle pressure. The reactor was heated to the established temperature for each reaction trial, and then the pressure was measured. Reaction was magnetically stirred keeping the established pressure.

After the appropriate reaction time, the reactor was cooled to room temperature and depressurized. The solvent was removed under vacuum and the final residue was analysed by ^{1}H NMR.

2) Method using urea.

In a typical experiment, monoamides (3 mmol), urea (1.2-5.8 mmol) and the desired amount of catalyst were introduced into the Schlenk flask equipped with a water-cooled condenser. The reaction mixture was magnetically stirred under vacuum (30 kPa) at 150°C for the appropriate reaction time. The reaction was cooled to room temperature. The product was purified by column chromatography on silica gel using CH_2Cl_2: MeOH as eluent. The final chromatographic fractions were analysed by ^{1}H NMR.

1.3.- EXPERIMENTAL DATA.

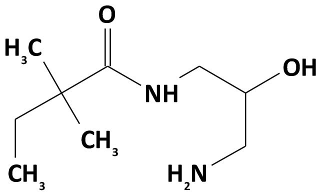

N-(3-Amino-2-hydroxypropyl)-2,2-dimethylbutanamide (2a) was synthesized as a colourless oil. Yield = 93%. ^{1}H NMR (400 MHz, $CDCl_3$)δ 6.14 (1 H, s), 3.60 – 3.50 (1 H, m), 3.32 (2 H, dddd, *J* = 25.7, 20.3, 9.4, 4.8), 2.65 (2 H, ddd, *J* = 19.9, 12.6, 5.8), 1.53 – 1.42 (2 H, q), 1.15 – 1.03 (6 H, m), 0.83 – 0.72 (3 H, t). ^{13}C (101 MHz, $CDCl_3$) δ 178.95, 71.18, 44.68, 43.16, 42.45, 33.82, 24.96, 9.20. IR (ATR/ν) 3342.03, 2966.95, 1632.45 .HRMS (ESI) m/z calcd. for $C_9H_{21}N_2O_2$ $[M+H]^+$ 189.1598 , found 189.1598.

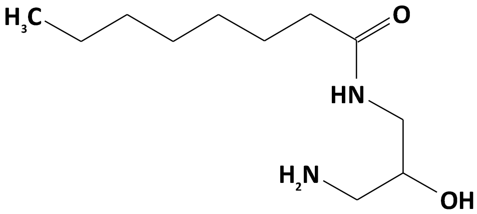

N-(3-Amino-2-hydroxypropyl)octanamide (2b) was synthesized as a white solid. Yield = 60%. M_p(°C)=114.8-119.0. ^{1}H NMR (400 MHz, CD_3OD) δ 3.67 (1 H, dtd, *J* =7.5, 5.8, 4.2), 3.27 – 3.17 (2 H, m), 2.67 (2 H, ddd, *J* =20.7, 13.2, 5.8), 2.23 – 2.16 (2 H, t), 1.64 – 1.55 (2 H, t), 1.36 – 1.25 (8 H, m), 0.90 (3 H, t, *J* =6.9).^{13}C NMR (101 MHz, CD_3OD) δ 175.51, 69.81, 43.55, 42.45, 35.60, 31.49, 28.92, 28.68, 25.55, 22.25, 12.99. IR (ATR/ν): 3354.57, 2916.81, 1639.2. HRMS (ESI) m/z calcd. for $C_{11}H_{25}N_2O_2$ $[M+H]^+$ 217.1911, found 217.1912.

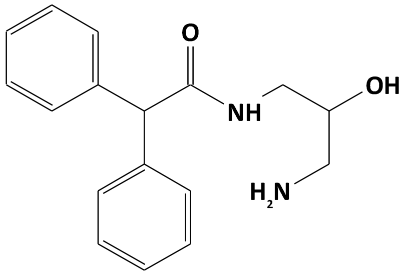

N-(3-Amino-2-hydroxypropyl)-2,2-diphenylacetamide (2c) was synthesized as a white power. Yield = 68%. M_p(°C)=99.7-103.8. ^{1}H NMR (400 MHz, $CDCl_3$) δ 7.37 – 7.21 (10 H, m), 6.10 (1 H, s), 4.92 (1 H, s), 3.60 (1 H, ddd, *J* =13.7, 6.9, 4.1), 3.36 (2 H, dddd, *J* =13.9, 11.9, 6.4, 3.9), 2.67 (2 H, ddd, *J* =19.8, 12.7, 5.7). ^{13}C NMR (101 MHz, $CDCl_3$) δ 172.74, 139.33, 128.81, 128.68, 127.20, 70.93, 59.23, 44.69, 43.51. IR (ATR/ν) 3315.97, 2924.23, 1644.12. HRMS (ESI) m/z calcd. for $C_{17}H_{21}N_2O_2$ $[M+H]^+$ 285.1598, found 285.1600.

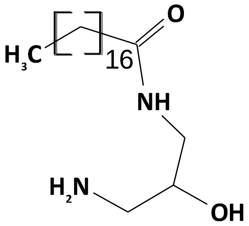

N-(3-Amino-2-hydroxypropyl)octadecanamide was synthesized as a white solid. Yield = 63%. M_p(°C)=114.9-118.1. ^{1}H NMR (400 MHz, CD_3OD) δ 3.65 – 3.56 (1 H, m), 3.26 – 3.14 (2 H, m), 2.61 (2 H, ddd, *J* =20.3, 13.2, 5.7), 2.19 (2 H, t, *J* =7.1), 1.59 (2 H, m), 1.30 (28 H, d, *J* =14.0), 0.89 (3 H, t, *J* =6.7). ^{13}C NMR (101 MHz, CD_3OD) δ 175.35, 70.53, 42.53, 35.68, 31.56, 29.26, 29.12, 28.94, 25.52, 22.21, 12.89. IR (ATR/ν): 3299.91, 2921.96, 1636.40. HRMS (ESI) m/z calcd. for $C_{21}H_{44}N_2O_2$ $[M+H]^+$ 357.3476, found 357.3472

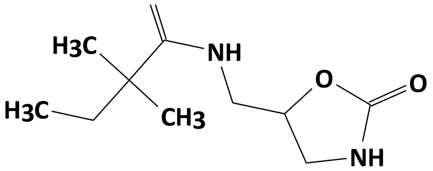

2,2-Dimethyl-N-[(2-oxo-1,3-oxazolidin-5-yl)methyl]butanamide was synthesized as a yellow oil.Yield = 3%. ^{1}H NMR (400 MHz, $CDCl_3$) δ 6.29 (1 H, t, *J* =5.9), 5.99 (1 H, s), 4.75 (1 H, tdd, *J* =9.6, 6.5, 3.2), 3.67 (2 H, dtd, *J* =12.4, 6.4, 3.4), 3.52 – 3.44 (1 H, m), 3.35 – 3.31 (1 H, m), 1.58 – 1.50 (2 H, q, *J* =7.4), 1.19 – 1.12 (6 H, m), 0.88 – 0.77 (3 H, t , *J* =7.4). ^{13}C NMR (101 MHz, $CDCl_3$) δ 178.67, 159.25, 75.82, 43.03, 42.59, 42.26, 33.73, 24.92, 24.90, 9.16. IR (ATR/ν): 3199.33, 2935.13, 1735.62. HRMS (ESI) m/z calcd. for $C_{18}H_{19}N_2O_3$ $[M+H]^+$ 311.1383, found. 311.1390.

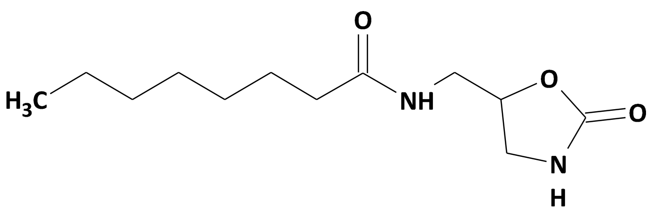

N-[(2-Oxo-1,3-oxazolidin-5-yl)methyl]octanamide was synthesized as a white solid. Yield = 10%. M_p(°C)=90.3-91.5, ^{1}H NMR (400 MHz, $CDCl_3$) δ 6.07 (1 H, s), 5.35 (1 H, s), 4.82 – 4.71 (1 H, m), 3.73 – 3.65 (2 H, m), 3.50 (1 H, dt, *J* 14.6, 6.2), 3.36 (1 H, ddd, *J*= 8.9, 6.6, 0.8), 2.21 (2 H, dd, *J* =16.2, 8.3), 1.63 (3 H, dd, *J*= 14.9, 7.5), 1.36 – 1.20 (9 H, m), 0.89 (3 H, dd, *J* =8.4, 5.5).^{13}C NMR (101 MHz, $CDCl_3$) δ 173.96, 158.99, 75.73, 42.92, 41.94, 36.59, 31.65, 29.20, 28.96, 25.65, 22.59, 14.05. IR (ATR/ν): 3305.39, 2922.59, 1752.01, 1726.94. HRMS (ESI) m/z calcd. for $C_{12}H_{22}N_2O_3Na$ $[M+Na]^+$ 265.1630, found.265.1677

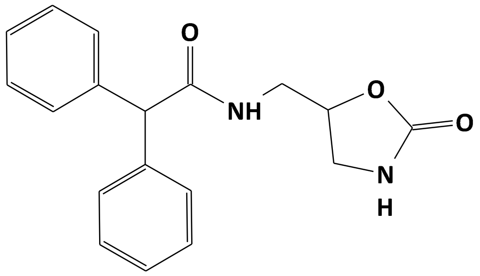

N-[(2-Oxo-1,3-oxazolidin-5-yl)methyl]-2,2-diphenylacetamide was synthesized as a white solid. Yield = 13%. M_p(°C)=174.2-175.1. ^{1}H NMR (400 MHz, D_6MSO) δ 8.62 (1 H, t, *J* =5.8), 7.47 (1 H, s), 7.34 – 7.18 (10 H, m), 5.02 (1 H, s), 4.63 – 4.52 (1 H, m), 3.47 (1 H, t, *J*= 8.8), 3.38 – 3.26 (2 H, m), 3.11 (1 H, dd, *J*= 8.6, 6.8). ^{13}C NMR (400 MHz, D_6MSO) δ 172.15, 158.95, 140.71, 140.64, 128.91, 128.87, 128.67, 127.06, 74.58, 56.69, 42.83, 42.20. IR (ATR/ν): 3262.97, 3084.58, 1743.33, 1649.80. HRMS (ESI) m/z calcd. for $C_{18}H_{19}N_2O_3$ $[M+H]^+$ 311.1370 found. 311.1390

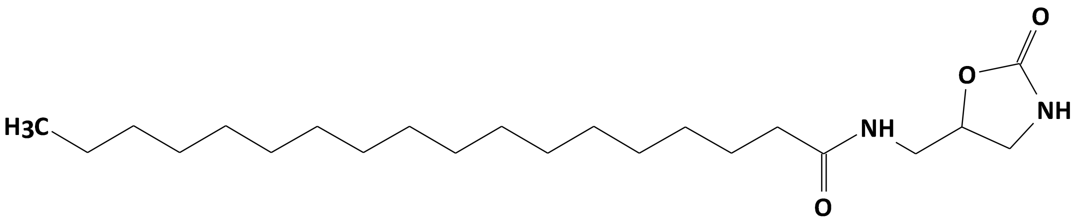

N-[(2-Oxo-1,3-oxazolidin-5-yl)methyl]octadecanamide was synthesized as a white solid. Yield = 8%. M_p(ºC)=116.7-118.2. ^{1}H NMR (400 MHz, $CDCl_3$) δ 5.90 (1 H, s), 3.55 (1 H, td, *J* =7.2, 3.7), 3.45 – 3.05 (1 H, m), 2.65 (1 H, ddd, *J* =20.1, 12.7, 5.8), 2.13 (1 H, t, *J* =7.7), 1.60 – 1.49 (1 H, m), 1.20 (7 H, d, *J* =13.4), 0.81 (1 H, t, *J* =6.9). ^{13}C NMR (101 MHz, $CDCl_3$) 172.8481, 160.9660, 77.30, 41.91,

36.62, 31.91, 30.91, 29.68, 29.64, 29.60, 29.46, 29.34, 29.30, 29.25, 25.64, 22.67, 14.10. IR (ATR/ν): 3296.71, 2916.81.96, 1729.83, 1640.16,. HRMS (ESI) m/z calcd. for $C_{22}H_{43}N_2O_3$ $[M+H]^+$ 383.3285, found 383.3268.

2.- SYNTHESIS OF TRIAZOLES.

Part of this was performed at the Warwick University under the supervision of the Prof David Haddleton

2.1.- REAGENTS AND EQUIPPMENT.

D-Mannose (99%, Sigma-Aldrich), L-fucose (98%, Sigma-Aldrich), D-glucose (99.5%, Sigma-Aldrich), propargyl alcohol (99%, Sigma-Aldrich, The H_2SO_4-silica catalyst was prepared according to the procedure described in the literature[41]. Sodium (+)-L-ascorbate (99%, Acros), $CuSO_4{\cdot}5H_2O$ (98%, Panreac.), glycerol (99.5%, Fischer Scientific), CTMS (98%, ACROS), acetic acid (98.5%, Panreac), caprylic acid (98%, Fluka), palmitic acid (98%, Aldrich), myristic acid (99%, Sigma), stearic acid (98%, Probus), sodium azide (99%, Aldrich), sodium hydroxide (97%, Sigma- Aldrich), acryloyl chloride (97%, Aldrich), 2-chloro-1,3-dimethylimidazolium chloride (DMC) (95%, Carbosynth), triethylamine (99.5%, Fluka), $CuBr_2$.(99%, Aldrich), ethyl α-bromoisobutyrate (EBiB) (98%, Alfa Aesar), tris[2-(dimethylamino)ethyl]amine (Me_6TREN)(98%, TCI), DMSO (99%, Fluka), HCl (37%, Panreac), copper wire (diameter=0.25 mm) which was pre-treated by washing in hydrochloric acid for 15 min and rinsed thoroughly with MiliQ water, dried under nitrogen and used immediately. Silica Gel 0,040-0.063 (Merk), Amberlite Resin IR-120H Hydrogen form (Aldrich), Membrane dialysis (1K MWCO) was obtained from Spectrum Laboratories.

The diazides (1b, 1d, 1f, 1h) and 1,3-dichloro-2-propanol (16) were synthesised from glycerol and the corresponding acid according the procedure previously described[92]. The reaction crude from diazides derivatives was not purified, due to their instability, and directly used for further reactions. Solvents

were purchased in Aldrich, Fluka and Across and were used without further purification, if it is not specified.

^{1}H and ^{13}C were recorded on Varian AS400 MERCURYplus (^{1}H, 400MHz and ^{13}C, 100 MHz), using CD_3OD, D_6MSO and D_2O as solvents. The spectra were recorded at 30°C and 20 s of relaxing time. The chemical shifts (δ) are reported in ppm relative to the solvent used. Spin multiplicities are reported as a singlet (s), doublet (d), or triplet (t) with coupling constants (J) given in Hz, or multiplet (m).

The melting points were measured by open capillary tubes in a Gallenkamp equipment. They are uncorrected.

IR spectra were recorded on a Jasco FT/IR-6300 equipment, in a range between 600 to 4000 cm^{-1}, prepared to take 60 spectras/second with a resolution of 16 cm^{-1} working with ATR and in a Bruker VECTOR-22 FTIR spectrometer using Golden Gate diamond attenuated total reflection cell.

SPR sensograms were recorded in a BioRad ProteOn XPR36 SPR biosensor (Biorad, Hercules CA). Soluble C-lectine ligands were immobilized to 6000 response units (RU) on discrete channels within Biorad GLC sensor chips. Soluble-phase analytes were prepared in 25mM HEPES pH 7.4, 150mM NaCl, 3mM $CaCl_2$, 0.05% TWEEN-20 and flowed over the immobilized materials at a rate of 25µL/min at 25ºC.

ESI-MS was recorded on a Thermo Finigan LQC Deca quadrupole ion trap mass spectrometer equipped with an atmospheric pressure ionization source operating in the nebulizer assisted electrospray mode and was used in positive ion mode. High resolution mass spectra were recorded by direct infusion to a mass spectrometer Agilent G6510AA Q-TOF using ESI ionization in positive ion mode and data was acquired in the 50-1000 *m/z* range. MALDI-TOF/TOF data was recorded on an Ultraflex III Bruker, with a DHB matrix with and without deflexion at 800 Da.

2.2.- METHODS.

2.2.1.-General procedure for the synthesis of propargyl sugar(Figure 28)[41].

In a typical experiment, a suspension of sugar (**10**-**12**) (70 mmol), propargyl alcohol (340 mmol) and H_2SO_4-silica (340 mg) was stirred at 65°C overnight. After cooling to room temperature, the reaction mixture was transferred to a silica gel column and eluted with CH_2Cl_2: MeOH (8: 1) or with CH_2Cl_2: MeOH (10: 1) for fucose derivative. The silica gel column allowed the elimination of the propargyl alcohol excess used in the reaction.

2.2.2.-General procedure for CuAAC Click reaction.

The reactions were carried out according the methodology previously described by *Zhang et al*.[33] with some modifications.

Synthesis of alkyne functional sugars. (Figure 28)

In a typical experiment a diazido derivative(2.1.3.2; Figure 8) (0.4 mmol) and propargyl sugar (1 mmol) were added into a MeOH: H_2O (2: 1, 12 mL) solution. An aqueous solution of $CuSO_4{\cdot}5H_2O$ (7.5%) and sodium (+)-L-ascorbate (10% w/w) were sequentially added. The mixture was stirred at room temperature for 24 h. Methanol was removed by vacuum and the residue mixture was freeze dried to remove water. The final green solid was purified by silica gel column chromatography using CH_2Cl_2: MeOH (10: 1) as eluent.
Table 1 shows the different derivate prepared, shown in Figure 28. Each of these solid compounds bear two sugar moieties.

Propargyl Sugar	Alkane Chain		
	**C8 Yield (%)**	**C14 Yield (%)**	**C16 Yield (%)**
**Glucose**	55.3	ns	44.2
**Mannose**	40.9	47.4	42.4
**Fucose**	54.5	ns	57.7

Table 1: *Yields corresponding to the* alkane derivates bearing two sugar moieties.

ns= no synthesized. * cannot be purified

Synthesis of D-Mannose glycomonomer (Figure 29).

In a typical experiment the azido sugar (1 mmol) and the alkyl ester (0.8 mmol) were added into a THF: H_2O (2: 1, 12 mL) solution at 50°C. An aqueous solution of hydroquinone (12%), $CuSO_4{\cdot}5H_2O$ (7.5%) and sodium (+)-L-

ascorbate (10% w/w) aqueous solutions were sequentially added. The mixture was stirred at 50°C for 24 h. THF was removed by vacuum and the residue mixture was freeze dried to remove the water. The final green solid was purified by silica gel column chromatography using CH_2Cl_2: MeOH (2: 1) as eluent.

2.2.3.- General procedure for the synthesis of D-mannopyranosyl azide

The reaction was carried out following a previously described procedure[44]. 2-Chloro-1,3-dimethylimidazolinium chloride (11.6 g, 68.6 mmol) was added to a solution of D-mannose (4.24 g, 23.5 mmol), triethylamine (32.5 mL, 233 mmol) and sodium azide (**20**) (12.16 g, 186.9 mmol) dissolved in water (110 mL). After stirring for 1 h at 0°C, the reaction mixture was let to react at room temperature overnight. After that, it was concentrated under vacuum and ethanol (200 mL) was added. The resulting solid was removed by filtration. The filtrate was concentrated under vacuum, the residue dissolved in water (70 mL) and the aqueous solution washed several times with dichloromethane. The aqueous solution was passed through a short column of acidic Amberlite® IR-120, previously activated with 1 M sodium hydroxide. The resulting aqueous solution was freeze-dried overnight to lead to the desired product.

2.2.4.- General procedure for the synthesis of 1,3-bis(prop-2-yn-1-yloxy)propan-2-ol .

The reaction was carried following a previously described methodology[93]. 1,3-Dichloropropan-2-ol (5.14g, 39 mmol) was added dropwise to a mixture of propargyl alcohol (9.0 mL, 155.7 mmol.) and sodium hydroxide (1.10 g, 27.6 mmol) in water (50 mL). The mixture was heated at reflux for 3 h, cooled and neutralized with 2M HCl. After extraction with CH_2Cl_2 (3 x 60 mL), the organic layers were dried and concentrated under vacuum. The residue was purified by silica-gel column chromatography (CH_2Cl_2) to lead the desired compound as a clear yellow oil.

2.2.5.- General procedure for the synthesis of 1,3-bis(prop-2-yn-1-yloxy)propan-2-yl prop-2-enoate.

The procedure was carried out following a general previously described procedure with slightly modifications[94]. 1,3-Bis(prop-2-yn-1-yloxy)propan-2-ol (1.8 g, 11 mmol) and Et_3N (2.0 mL, 14.3 mmol) were dissolved in 80 mL of CH_2Cl_2 anhydrous and the solution was cooled at 0°C under argon atmosphere. A solution of acryloyl chloride (1.5 mL, 18.5 mmol) in CH_2Cl_2 (15 mL) was added dropwise. The resulting mixture was stirred overnight at room temperature, then poured into 50 mL of cold water, and finally extracted with CH_2Cl_2 (3 x 50 mL). The combined organic phases were dried over Na_2SO_4. The solvent was removed under vacuum. The product was obtained as a yellow oil with enough purity, which led to the desired compound without any further purification needed.

2.2.6.- General procedure fo the homopolymerization of D-mannose acrylate glycomonomer via SET-LRP (Figure 30).

The procedure was carried out following a general previously described procedure[33].

All polymerizations were carried out using standard Schlenk techniques under an inert atmosphere of oxygen-free nitrogen. Mannose glycomonomer (1mmol), $CuBr_2$ (0.01 mmol) and DMSO (3 mL) were added to a Schlenk tube fitted with a magnetic stir bar and a rubber stopper. Nitrogen was bubbled into the mixture for 15 min. Pre-degassed Me_6TREN (0.018 mmol) and EBiB (0.1 mmol) were sequentially added via gas tight syringe. Finally, pre-activated copper wire was carefully added under nitrogen protection. The Schlenk tube was sealed and the light green solution was allowed to polymerize at 25°C. Samples of the reaction mixture were carefully taken at suitable time periods for analysis. The sample for ^{1}H NMR analysis was directly diluted with D_6MSO. After 24 h, the reaction was stopped via exposure to the air and the mixture was diluted with water and dialysed against water for two days. Finally, the glycopolymer was recovered by freeze drying.

2.3.- EXPERIMENTAL DATA.

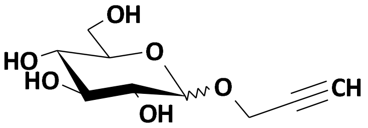

D-Glucopyranoside, 2-propyn-1-yl was obtained as an yellow oil after drying under vacuum. Yield = 53% (5: 1 in α: ß ratio). ^{1}H NMR (400 MHz, CD_3OD) δ 5.00 (1 H, d, *J*=5.9), 4.49 – 4.45 (0.1 H, d, *J* =1.3), 4.32 (0.9 H, d, *J* =1.3), 3.91 – 3.27 (6 H, m, residues of glucose), 2.87 (1 H, d, *J* =2.3). ^{13}C NMR(101 MHz, CD_3OD) δ 102.13, 98.62, 78.03, 75.00, 74.89, 74.11, 73.32, 71.67, 71.62, 62.75, 62.53, 56.57, 55.25. IR (ATR/ν): 3370.96, 2938.02, 2117.46. ESI-MS *m/z*: calcd for $C_9H_{14}O_6Na$ $[M+Na]^+$, 241.1; found 240.9.

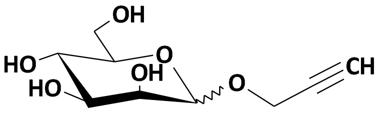

D-Mannopyranoside, 2-propyn-1-yl was obtained as an yellow oil after drying under vacuum. Yield=42% (4: 1 in α: ß ratio). ^{1}H NMR (400 MHz, CD_3OD) δ 4.86 (1 H, s), 4.33 (0.2 H, d, *J* =2.3), 4.18 (0.8 H, d, *J* =2.4), 3.82 – 3.34 (6 H, m, residues of mannose), 2.77 (0.2 H, t, *J* =2.4), 2.75 (0.8 H, t, *J*= 2.4). ^{13}C NMR (101 MHz, CD_3OD) δ 99.85, 99.34, 78.49, 75.26, 75.11, 72.51, 72.39, 72.04, 68.49, 62.84, 56.37, 54.84.IR (ATR/ν): 3361.32, 2942.84, 2117.46. ESI-MS *m/z*: calcd. for $C_9H_{14}O_6Na$ $[M+Na]^+$, 241.1; found 240.9.

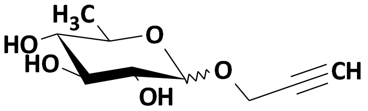

D-Fucopyranoside, 2-propyn-1-yl was obtained as an yellow oil after drying under vacuum. Yield=57% (5: 2 in α: ß ratio) ^{1}H NMR (400 MHz, CD_3OD) δ 4.96 (0.15 H, d, *J* =3.4), 4.38 (0.85 H, t, *J*=1.9), 4.28 (1 H, d, *J*=2.4), 4.02 – 3.29 (7 H, m, residues of fucose), 1.29 (0.15 H, d, *J*=6.4), 1.23 (0.85 H, d, *J* 6.6). ^{13}C NMR (101 MHz, CD_3OD) δ 102.53, 99.29, 75.11, 73.63, 72.07, 71.58, 69.77, 68.04, 56.36, 55.55, 16.51. IR (ATR/ν): 3266.82, 2931.27, 2119.39. ESI-MS *m/z*: calcd. for $C_9H_{14}O_6Na$ $[M+Na]^+$, 225.1; found 224.9.

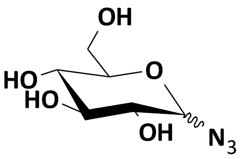

D-Mannopyranosyl azide was obtained as a white solid. M_p(°C)= decomposes at 141.5°C before melt. Yield = 77%. ^{1}H NMR (400 MHz, D_2O) δ 5.32 (1H, d, *J* =5.6), 3.81 – 3.48 (6H, m, residues of mannose). ^{13}C NMR (101 MHz, D_2O) δ 89.68, 74.58, 69.77, 69.71, 66.33, 60.76. IR (ATR/ν): 3424.96; 3293.82; 2960.20, 2128.06. The NMR signals correspond to a previously described compound[95, 96]

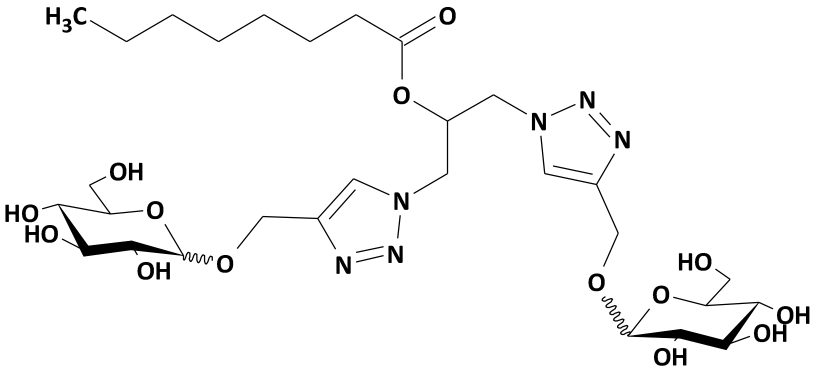

1-(4-(D- Glucopyranosyl)-1H-1,2,3-triazol-1-yl)-3-(4-(D- Glucopyranosyl)-1H-1,2,3-triazol-1-yl)propan-2-yl octanoate was obtained as a white solid, Yield = 55%, ^{1}H NMR (400 MHz, CD_3OD) δ 8.13 – 8.03 (2 H, s), 5.74 – 5.65 (1 H, m), 4.74 – 4.63 (4 H, m), 3.98 – 3.35 (12 H, m), 2.27 (2 H, t, *J* =7.4), 1.51 – 1.42 (2 H, m), 1.38 – 1.14 (8 H, m), 0.91 (3 H, t, *J* =6.9). ^{13}C NMR (101 MHz, CD_3OD) δ 173.73, 145.82, 126.52, 103.47, 99.56, 78.07, 78.00, 75.06, 74.07, 73.49, 71.82, 71.65, 71.22, 62.74, 61.33, 51.67, 34.67, 32.83, 30.01, 25.70, 23.69. IR (ATR/ν): 3329.45, 2924.10, 1739.29, 1638.39, 1227.22, 1019.64. ESI-MS *m/z*: calcd. for $C_{29}H_{48}N_6O_{14}Na$ $[M+Na]^+$, 727.3; found 727.3.

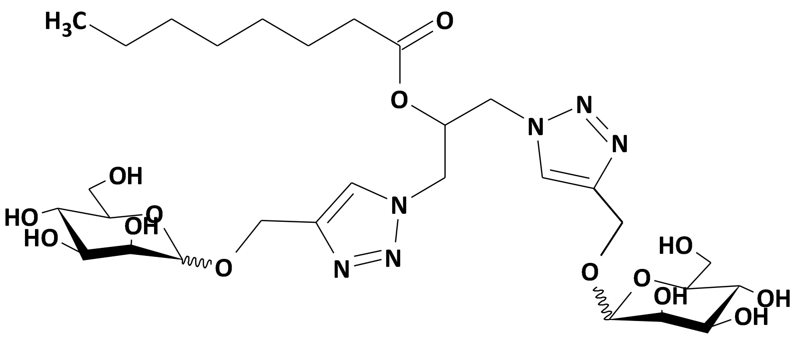

1-(4-(D- Mannopyranosyl)-1H-1,2,3-triazol-1-yl)-3-(4-(D- Mannopyranosyl)-1H-1,2,3-triazol-1-yl)propan-2-yl octanoate was obtained as a white solid, Yield = 41%, ^{1}H NMR (400 MHz, CD_3OD) δ 7.98 (2 H, s), 5.59 (1 H, dt, *J*= 11.0, 3.6), 4.70 – 4.44 (4 H, m), 3.80 – 3.40 (12 H, m), 2.19 – 2.06 (2 H, m), 1.38 – 1.27 (2 H, m), 1.25 – 1.01 (8 H, m), 0.80 (3 H, t, *J*= 6.9). ^{13}C NMR (101 MHz, CD_3OD) δ 173.73, 145.62, 126.53, 100.79, 75.04, 72.54, 72.03, 71.17, 68.64, 63.02, 60.62, 51.68, 34.66, 32.82, 30.02, 25.71, 23.69. IR (ATR/ν): 3330.45, 2925.10, 2855.99, 1738.87, 1639.45, 1227.43, 1024.56. ESI-MS *m/z*: calcd. for $C_{29}H_{48}N_6O_{14}Na$ $[M+Na]^+$, 727.3; found 727.3.

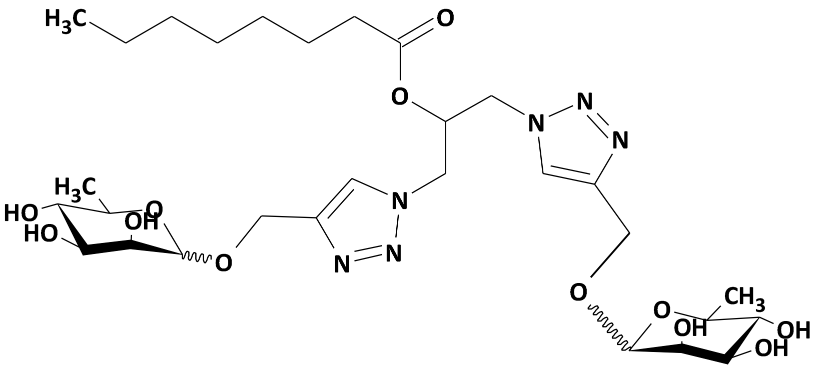

1-(4-(D- Fucopyranosyl)-1H-1,2,3-triazol-1-yl)-3-(4-(D- Fucopyranosyl)-1H-1,2,3-triazol-1-yl)propan-2-yl octanoate was obtained as white solid, Yield = 55%, ^{1}H NMR (400 MHz, CD_3OD) δ 8.07 (2 H, s), 5.69 (1 H, dt, *J* =11.2, 3.8), 4.81 (2 H, s), 4.67 (4 H, dd, *J* =16.7, 4.9), 4.02 – 3.65 (12 H, m), 2.26 (2 H, t, *J* =7.4), 1.51 – 1.41 (2 H, m), 1.31 – 1.12 (8 H, m), 0.92 (3 H, t, *J* =6.9). ^{13}C NMR (101 MHz, CD_3OD) δ 146.03, 126.29, 100.12, 73.63, 71.63, 71.22, 69.95, 67.89, 61.60, 51.63, 34.66, 32.84, 30.06, 25.70, 16.66. IR (ATR/ν): 3355.71, 2926.05, 1739.78, 1650.91, 1225.62, 1035.23. ESI-MS *m/z*: calcd. for $C_{29}H_{48}N_6O_{12}Na$ $[M+Na]^+$, 695.3; found 695.3.

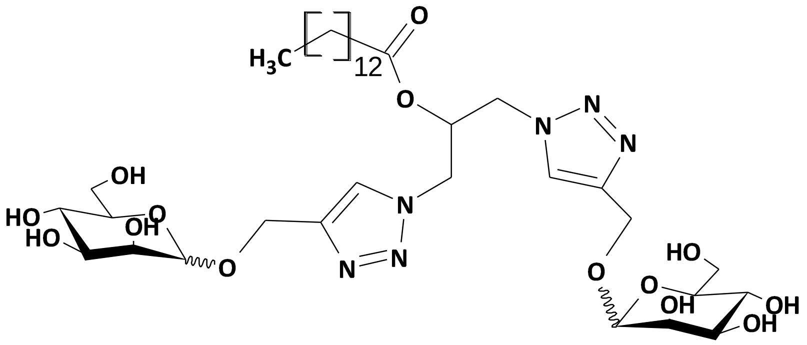

1-(4-(D- Mannopyranosyl)-1H-1,2,3-triazol-1-yl)-3-(4-(D- Mannopyranosyl)-1H-1,2,3-triazol-1-yl)propan-2-yl tetradecanoate was obtained as a white solid, Yield = 47%, M_p(°C)= 64.5-65.2. ^{1}H NMR (400 MHz, CD_3OD) δ 8.07 (2 H, s), 5.68 (1 H, dt, *J*= 11.2, 3.7), 4.81 – 4.57 (4 H, m), 3.88 – 3.49 (12 H, m), 2.22 (2 H, t, *J* =7.4), 1.43 (2 H, dt, *J* =14.7, 7.3), 1.30 (20 H, d, *J* =14.3), 0.89 (3 H, t, *J*= 6.8). ^{13}C NMR (101 MHz, CD_3OD) δ 172.52, 127.73, 126.70, 99.33, 73.57, 71.09, 70.58, 67.19, 61.56, 59.18, 50.23, 48.22, 48.01, 33.23, 31.65, 29.40, 29.38, 29.35, 29.17, 29.06, 28.93, 28.61, 24.28, 22.31, 13.03. IR (ATR/ν): 3330.46, 2925.48, 1739.48. HRMS (ESI) m/z calcd. for $C_{37}H_{65}N_6O_{14}$ $[M+H]^+$ 817.4528, found 817.4486.

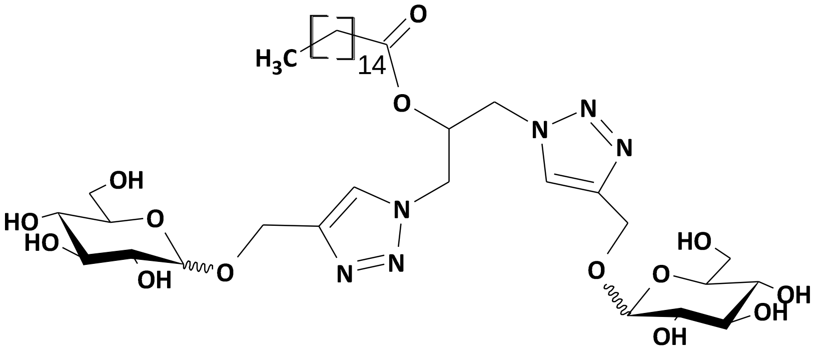

1-(4-(D- Glucopyranosyl)-1H-1,2,3-triazol-1-yl)-3-(4-(D- Glucopyranosyl)-1H-1,2,3-triazol-1-yl)propan-2-yl hexadecanoate was obtained as a yellow solid, Yield = 44%, M_p(°C)= 171.1-189.9. ^{1}H NMR (400 MHz, CD_3OD) δ 8.11 – 8.06 (2 H, s), 5.68 (1 H, dt, *J* =11.2, 3.7), 4.89 (2 H, dd, *J* =9.1, 3.6), 4.71 – 4.63 (4 H, m), 3.94 – 3.19 (12 H, m), 2.25 (2 H, t, *J*= 7.4), 1.45 (2 H, dt, *J*= 14.6, 7.4), 1.38 – 1.12 (24 H, m), 0.90 (3 H, t, *J*= 6.9). ^{13}C NMR (101 MHz, D_2O) δ 172.93, 143.79, 125.68, 97.75, 73.16, 71.95, 71.20, 69.41, 60.60, 31.94, 29.94, 29.74, 29.41, 29.04, 24.54, 22.61, 13.86. IR (ATR/ν): 3346.85, 2922.59, 1741.41. HRMS (ESI) m/z calcd. for $C_{37}\ H_{65}\ N_6\ O_{14}$ $[M+H]^+$ 817.4528, found 817.4553.

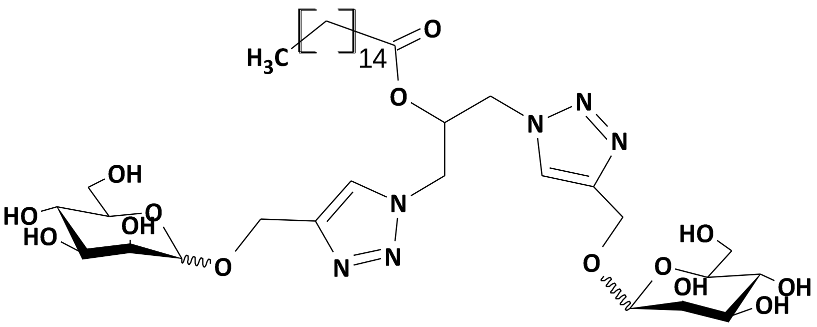

1-(4-(D- Mannopyranosyl)-1H-1,2,3-triazol-1-yl)-3-(4-(D- Mannopyranosyl)-1H-1,2,3-triazol-1-yl)propan-2-yl hexadecanoate, was obtained as a yellow solid, M_p(°C)= 156.4-157.9. Yield = 42%, ^{1}H NMR (400 MHz, CD_3OD) δ 8.08 (2 H, s), 5.69 (1 H, m), 4.65 (4 H, dd, *J* =13.2, 5.5), 3.96 – 3.48 (12 H, m), 2.26 – 2.14 (2 H, m), 1.48 – 1.38 (2 H, m), 1.36 – 1.08 (24 H, m), 0.89 (3 H, t, *J* =6.8). ^{13}C NMR (101 MHz, CD_3OD) δ 172.32, 172.29, 99.30, 73.56, 71.09, 70.57, 69.71, 67.18, 61.54, 59.24, 50.28, 33.25, 31.66, 29.41, 29.37, 29.20, 29.07, 28.96, 28.63, 24.30, 22.33, 13.08. IR (ATR/ν): 3360.35, 2921.63, 1741.41. HRMS (ESI) m/z calcd. for C_{37} H_{65} N_6 O_{14} $[M+H]^+$ 789.4241, found 789.4240.

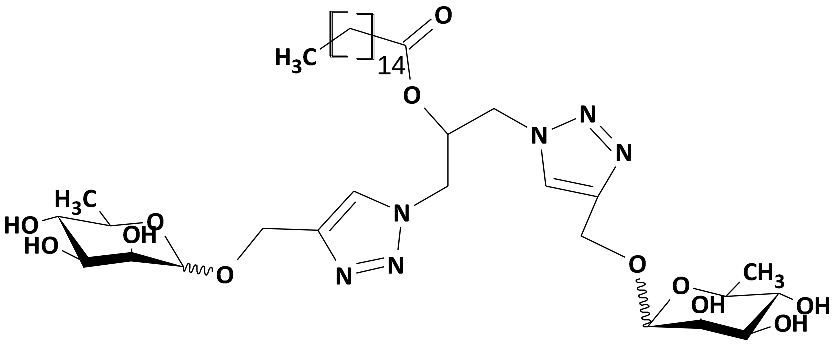

1-(4-(D- Fucopyranosyl)-1H-1,2,3-triazol-1-yl)-3-(4-(D- Fucopyranosyl)-1H-1,2,3-triazol-1-yl)propan-2-yl hexadecanoate, was obtained as a yellow solid, Yield = 58%, M_p(°C)= 50.8-51.9. ^{1}H NMR (400 MHz, CD_3OD) δ 8.07 (2 H, s), 5.68 (1 H, dd, *J* =7.4, 3.7), 4.77 (2 H, d, *J*= 3.3), 4.71 – 4.58 (4 H, m), 4.00 – 3.59 (12 H, m), 2.26 – 2.17 (2 H, m), 1.43 (2 H, dt, *J* 14.9, 7.4), 1.39 – 1.24 (24 H, m), 0.94 – 0.83 (3 H, m). ^{13}C NMR (101 MHz, CD_3OD) δ 172.26, 144.56, 124.83, 98.65, 72.18, 70.17, 68.50, 66.43, 60.13, 50.18, 33.38, 33.23, 31.65, 29.39, 29.34, 29.30, 29.25, 29.19, 29.14, 29.05, 29.04, 28.97, 28.94, 28.75, 28.64, 24.60, 24.27, 22.31, 15.24, 13.02. IR (ATR/ν): 3360.35, 2922.59, 1741.41. HRMS (ESI) m/z calcd. for C_{37} H_{65} N_6 O_{12} $[M+H]^+$ 785.4707, found 785.4655.

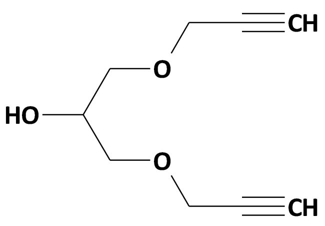

1,3-Bis(prop-2-yn-1-yloxy)propan-2-ol was obtained as an yellow oil. Yield = 18%, ^{1}H NMR (400 MHz, $CDCl_3$) δ 4.15 (1 H, q, *J* =2.5), 3.65 – 3.50 (8 H, m), 2.48 – 2.45 (2 H, m). ^{13}C NMR (101 MHz, $CDCl_3$) δ 79.14, 75.20, 70.60, 70.07, 58.61. IR (ATR/ν): 3411.46; 3291.89, 2921.63, 2116.49. ESI-MS *m/z*: calcd. for $C_9H_{12}O_3Na$ $[M+Na]^+$, 191.1; found 191.0.

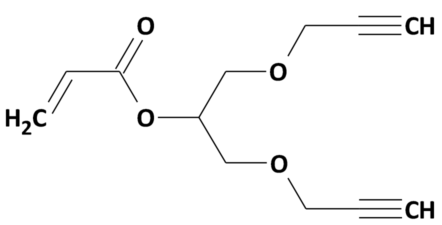

1,3-Bis(prop-2-yn-1-yloxy)propan-2-yl prop-2-enoate was obtained as an yellow oil, Yield = 95%. ^{1}H NMR (400 MHz, CD_3OD) δ 6.47 – 6.34 (1 H, m), 6.22 – 6.11 (1 H, m), 5.89 (1 H, d, *J* 10.4), 5.22 – 5.14 (1 H, m), 4.17 (4 H, dd, *J* =7.8, 2.3), 3.77 – 3.62 (4 H, m), 2.82 (2 H, dt, *J* =8.8, 2.4).^{13}C NMR (101 MHz, CD_3OD) δ 165.64, 130.42, 128.07, 78.87, 74.71, 71.45, 67.94, 57.86, 48.23, 48.02, 47.81, 47.59, 47.38. IR (ATR/ν): HRMS (ESI) m/z calcd. for C_{12} H_{15} O_4 $[M+H]^+$ 223.0962, found 223.0965.

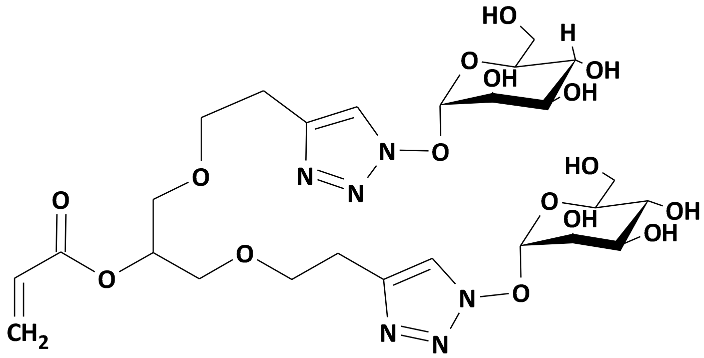

D-Mannose glycomonomer, was obtained as am yellow sticky oil. Yield = 24%. ^{1}H NMR (400 MHz, CD_3OD) δ 8.18 – 8.08 (2 H, m), 6.38 (1 H, dd, *J*=17.3, 1.5), 6.14 (1 H, dd, *J*=17.3, 10.4), 6.01 (1 H, dd, *J*=9.9, 1.6), 5.89 (1 H, dd, *J*=10.4, 1.5), 5.21 – 5.12 (1 H, m), 4.65 (4 H, dt, *J*=29.8, 7.8), 3.83 – 3.25 (mannose residues + 4H). ^{13}C NMR (101 MHz, CD_3OD) δ 165.65, 144.53, 130.68, 128.01, 123.69, 86.95, 77.17, 71.53, 71.13, 68.66, 68.43, 67.79, 67.15, 63.60, 61.09. IR (ATR/ν): 3339.14, 2928.38, 2115.53, 2070.21, 1716.34. HRMS (ESI) m/z calcd. for $C_{24}H_{35}N_6O_{14}$ $[M+H]^+$ 631.2227, found 631.2217.

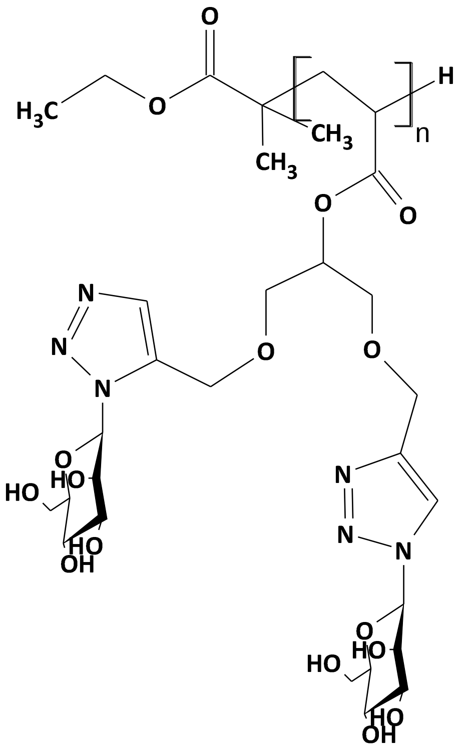

Mannose polymer was obtained as a white solid. ^{1}H NMR (400 MHz, D_2O) 8.11 (broad band), 6.00 (broad band), 4.02 (broad band), 3.68 (broad band), 3.19 (broad band), 1.94 – 1.49 (broad band).IR (ATR/ν): 3337.21, 2933.20, 1719.23.

MALDI-ToF m/z calc for DP=2 $C_{54}H_{84}N_{12}Na_1O_{30}$ $[M+Na]^+$ calcd. 1403.5, found 1403.5; DP=3 $C_{78}H_{120}N_{18}Na_1O_{44}$ $[M+Na]^+$ calcd. 2035.8, found 2035.8; DP=4 $C_{102}H_{156}N_{24}Na_1O_{58}$ $[M+Na]^+$ calcd. 2668.0, found 2667.9; DP=5 $C_{126}H_{192}N_{30}Na_1O_{72}$ $[M+Na]^+$ calcd. 3300.2, found 3300.4

MALDI-ToF analysis performed to characterize the structure of the glycopolymer. Peaks at 1403, 2035, 2668 and 3301 Da (*m*/*z*) cationised with Na^+ were attributed to dead polymer chains with terminal hydrogen. with 2 to 5 chains added,respectively (Scheme 4.14). The exchange of the bromine for the proton were mainly caused by disproportionation and chain transfer side reaction and thus leading to lose of terminal bromine. It must be note that there appears also repited not identified signals at +34, +58, +106 m/z. Hence, the polymer with terminal bromine was not detected using the MALDI-ToF technique.

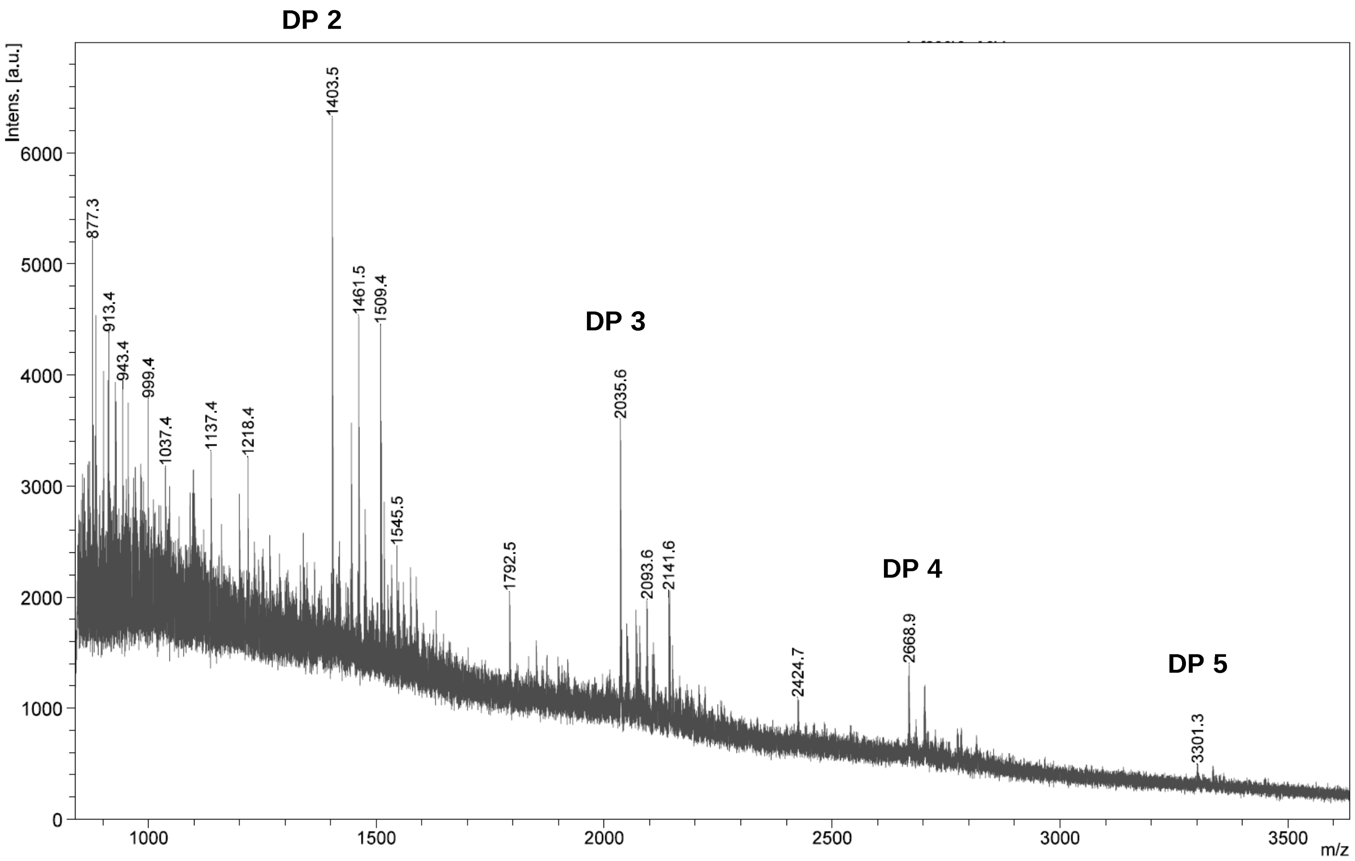

Figure 4.3: MALDI-ToF spectra of the glycopolymer (**31k**) obtained by SET-LRP.
